# The contribution of community-based conservation models to conserving large herbivore populations

**DOI:** 10.1038/s41598-024-66517-9

**Published:** 2024-07-13

**Authors:** Christian Kiffner, Charles A. H. Foley, Derek E. Lee, Monica L. Bond, John Kioko, Bernard M. Kissui, Alex L. Lobora, Lara S. Foley, Fred Nelson

**Affiliations:** 1 Junior Research Group Human-Wildlife Conflict and Coexistence, Leibniz Centre for Agricultural Landscape Research (ZALF), Müncheberg, Germany; 2 The School for Field Studies, Centre For Wildlife Management Studies, PO Box 304, Karatu, Tanzania; 3 Department of Land Use & Governance, Humboldt-University of Berlin, Berlin, Germany; 4 Tanzania Conservation Research Program, Lincoln Park Zoo, Chicago, IL USA; 5 Wild Nature Institute, Concord, NH USA; 6 Department of Evolutionary Biology and Environmental Studies, University of Zurich, Zurich, Switzerland; 7 Tanzania Wildlife Research Institute (TAWIRI), Arusha, Tanzania; 8 Maliasili, Essex Junction, VT USA

**Keywords:** Conservation effectiveness, Community-based conservation, Population dynamics, Social-ecological systems, Fortress conservation, Conservation biology, Population dynamics

## Abstract

In East Africa, community-based conservation models (CBCMs) have been established to support the conservation of wildlife in fragmented landscapes like the Tarangire Ecosystem, Tanzania. To assess how different management approaches maintained large herbivore populations, we conducted line distance surveys and estimated seasonal densities of elephant, giraffe, zebra, and wildebeest in six management units, including three CBCMs, two national parks (positive controls), and one area with little conservation interventions (negative control). Using a Monte-Carlo approach to propagate uncertainties from the density estimates and trend analysis, we analyzed the resulting time series (2011–2019). Densities of the target species were consistently low in the site with little conservation interventions. In contrast, densities of zebra and wildebeest in CBCMs were similar to national parks, providing evidence that CBCMs contributed to the stabilization of these migratory populations in the central part of the ecosystem. CBCMs also supported giraffe and elephant densities similar to those found in national parks. In contrast, the functional connectivity of Lake Manyara National Park has not been augmented by CBCMs. Our analysis suggests that CBCMs can effectively conserve large herbivores, and that maintaining connectivity through CBCMs should be prioritized.

## Introduction

Populations of large herbivores in East African savanna and grassland ecosystems move widely through landscapes where the distribution of forage and water vary considerably across space and time, resulting in seasonal animal migrations^1^. These herbivore populations in East Africa have declined markedly during the last decades^2^ both inside and outside fully protected areas^3–5^ as a result of unsustainable legal and illegal hunting^6^, deterioration of rangelands^7^, and loss and fragmentation of habitat due to expansion of agriculture and infrastructure^8,9^. Acknowledging that government protected areas alone are insufficient as a single measure to halt or reverse wildlife declines and to create landscapes that support both people and wildlife^10^, a diverse set of community-based conservation models (CBCMs) has been implemented in several parts of East Africa. In practice, these CBCMs often augment existing protected area networks and safeguard critical habitats on community and private lands^11,12^. In ecosystems that still sustain long distance migrations of large herbivores, CBCMs can provide suitable and safe habitat for wildlife and can contribute to effective conservation of migratory populations^1,13^. CBCMs in the region often aim for sustainable coexistence between wildlife and the livestock of pastoralist communities, as well as the Indigenous communities’ rangeland management practices, which include seasonal grazing reserves and rules for pasture access^14,15^.

Testing the effectiveness of conservation interventions is a key topic in conservation biology^16^, yet frequently hampered by a lack of monitoring data in CBCMs^17,18^. The few site-specific assessments of the ecological effectiveness of different Tanzanian CBCMs indicate mixed results, including wildlife population declines, stable population trends, and marked population size increases^19–24^. Ecological assessments of CBCMs are often solely based on temporal trends of target species in one study area^25^ or on comparisons with density estimates in either human-dominated^20,21,26^ or fully protected areas^22^. While valuable for informing local management in the short-term, such site-specific monitoring efforts only partially inform whether CBCMs effectively contribute to conserving wildlife populations at the ecosystem scale. This is because the distribution of large mammals in heterogeneous savanna ecosystems is dynamic^27–29^ with local population sizes affected by animal movement^30^. To more accurately assess the ecological effectiveness of CBCMS, it is beneficial to compare wildlife densities across multiple management units, including positive reference points such as national parks (while keeping in mind that they are not entirely pristine^31,32^), and areas with minimal conservation efforts.

**Figure 1:**
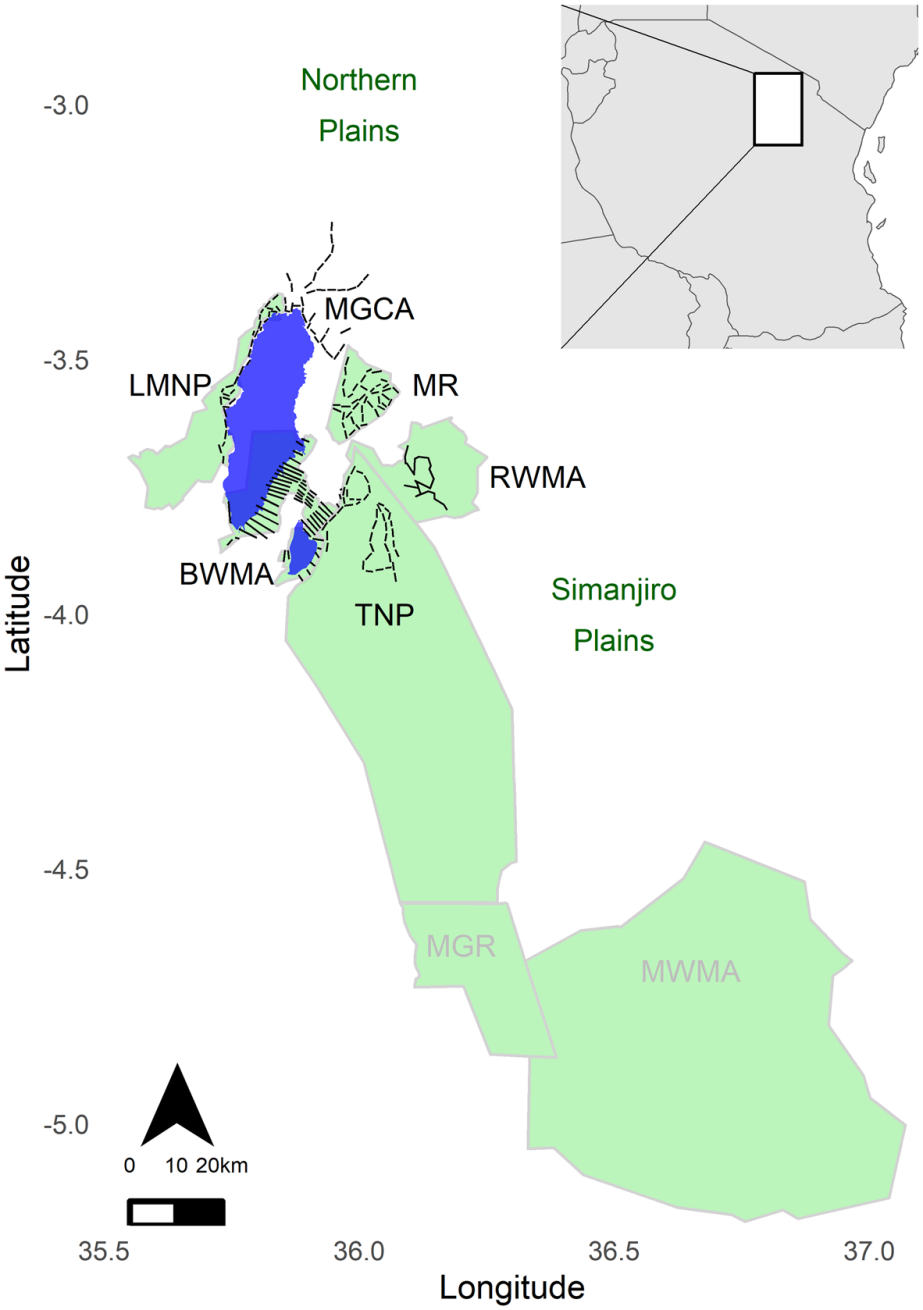
Map of the Tarangire Ecosystem in northern Tanzania; the inset in the top right indicates the location of the ecosystem within Tanzania. Terrestrial line transects (black lines) were carried out in Lake Manyara (LMNP) and Tarangire National Park (TNP), Burunge (BWMA) and Randilen Wildlife Management Area (RWMA), Manyara Ranch (MR), and the Mto wa Mbu Game Controlled Area (MGCA, no boundary data available). Mkungunero Game Reserve (MGR) and Makame Wildlife Management Area (MWMA). ‘Northern Plains’ and ‘Simanjiro Plains’ denote approximate locations of the wet season ranges of zebra (*Equus quagga*) and wildebeest (*Connochaetes taurinus*). For completeness, we also mapped Mkungunero Game Reserve (MGR) and Makame Wildlife Management Area (MWMA) which are part of the TE but not sampled. Blue polygons are alkaline lakes. We created both maps (study area within Tanzania and details of our study area) using the 'ggplot2' (version 3.3.6), 'rgdal' (version 1.5–23), and 'ggsn' (version 0.5.0) packages in R (version 4.2.2)^70^. Maps are based on open source (area polygons, lakes) and our own (transects) shapefiles.

The fragmented Tarangire Ecosystem of northern Tanzania (Fig. 1) maintains one of the last remaining long-distance migrations of large herbivores in Africa^1,33^. Compared to historical baselines, wildlife population sizes have declined substantially inside and outside of protected areas, with particularly pronounced declines during the 1980s and 1990s^31,34,35^. During the last two decades, three strategically placed community-based conservation areas [Burunge and Randilen Wildlife Management Area (WMA), and Manyara Ranch] were established in parts of the ecosystem^36^, mostly around Tarangire National Park (NP), to counteract these declines. These community-based conservation areas protect specific habitats for wildlife while allowing limited human activity. Some CBCMs allow seasonal livestock grazing in specified areas, some allow regulated hunting, and they typically employ game scouts to enforce wildlife laws and community land-use regulations. The type and intensity of natural resource utilization and the degree of community involvement in governance differ by CBCM category (see Table 1).

**Table 1:** Key information on management practices and monitoring efforts for each considered management unit in the Tarangire Ecosystem, northern Tanzania.

Management unit	Overall conservation model	Hunting regulations	Livestock management	Wildlife monitoring efforts
Tarangire National Park (positive control)	Top down management by employees of Tanzania National Parks; state-funded conservation; main revenue: income from photographic tourism	No hunting allowed. Enforcement implemented by rangers employed by Tanzania National Parks	No livestock allowed. Enforcement implemented by rangers employed by Tanzania National Parks	Surveys were conducted by driving along road transects with open-top vehicles. A total of 24 seasonal surveys were carried out from October 2011 to October 2019
Lake Manyara National Park (positive control)	Top down management by employees of Tanzania National Parks; state-funded conservation; main revenue: income from photographic tourism	No hunting allowed. Enforcement implemented by rangers employed by Tanzania National Parks	No livestock allowed. Enforcement implemented by rangers employed by Tanzania National Parks	Surveys were conducted by driving along road transects with open-top vehicles. A total of 24 seasonal surveys were carried out from November 2011 to November 2019
Burunge wildlife management area (community-based conservation model CBCM)	Community-based conservation, managed by elected council members from WMA villages; main revenue: income from photographic tourism and donations from non-governmental organizations	The western section initially contained a hunting block. However, since 2014, allocated quotas are not realized and the hunting block is managed for photographic tourism. Enforcement is implemented by village game scouts who are typically residents of the member villages, employed by the Wildlife Management Area	The area is structured into distinct management zones. No livestock is allowed in areas dedicated to wildlife	Surveys were conducted by driving or walking along systematically distributed transects. A total of 7 seasonal surveys were carried out from September 2011 to July 2018
Randilen wildlife management area (community-based conservation model CBCM)	Community-based conservation, managed by elected council members from WMA villages; main revenue: income from photographic tourism and donations from non-governmental organizations	No hunting allowed. Enforcement is implemented by village game scouts who are typically residents of the member villages, and employed by the Wildlife Management Area	The area is structured into distinct management zones. No livestock is allowed in areas dedicated to wildlife	Surveys were conducted by driving along road transects with open-top vehicles. A total of 12 seasonal surveys were carried out from January 2012 to October 2015
Manyara Ranch (community-based conservation model CBCM)	Conservancy: Community-based conservation, managed by employees of the conservancy with input from advisory board which includes representatives from member villages; main revenue: donations from non-governmental organizations, supplemented with income from photographic tourism and livestock	No hunting allowed. Enforcement is implemented by game scouts who are typically residents of the member villages, and employed by a non-governmental organization	Manyara Ranch owns its own cattle and sheep herds which are managed using a grazing rotation. During the dry season, herders from member villages are permitted to graze their own livestock in specified areas of the ranch	Surveys were conducted by driving along road transects with open-top vehicles. A total of 24 seasonal surveys were carried out from November 2011 to November 2019
Mto wa Mbu game controlled area (negative control)	Top down management by employees of Tanzania Wildlife Authority; state-funded conservation; main revenue: income from hunting blocks and photographic tourism	The northern section of the area contains a hunting block where trophy hunting is allowed. Hunting is limited though a quota system. Hunting restrictions are enforced by the Tanzania Wildlife Authority	High densities of livestock, grazing is largely unregulated	Surveys were conducted by driving along road transects with open-top vehicles. A total of 24 seasonal surveys were carried out from November 2011 to November 2019

Here, we test how these conservation efforts affected site-specific population dynamics of four wide-ranging, abundant, and functionally important wildlife species: African savanna elephant (*Loxodonta africana*), Masai giraffe (*Giraffa tippelskirchi*), plains zebra (*Equus quagga*), and wildebeest (*Connochaetes taurinus*). Wildlife populations were estimated from line distance sampling carried out seasonally from 2011 to 2019 in Burunge WMA, Randilen WMA, and Manyara Ranch. As spatial reference points, we considered population densities and associated trends in two fully protected areas: Tarangire NP, which is buffered by the considered CBCMs and Lake Manyara NP, which is not directly bordered by CBCMs. In addition, as counterfactual, we considered wildlife population trends in an area with few restrictions on human land use, Mto wa Mbu Game Controlled Area (GCA) (Fig. 1). To estimate area-specific annual trends, we fitted generalized additive models to seasonal density data and used a two-stage Monte Carlo simulation approach which propagates uncertainties from both the distance sampling estimates and trend analysis.

## Results

### Area-specific trends of large herbivore populations

**Figure 2:**
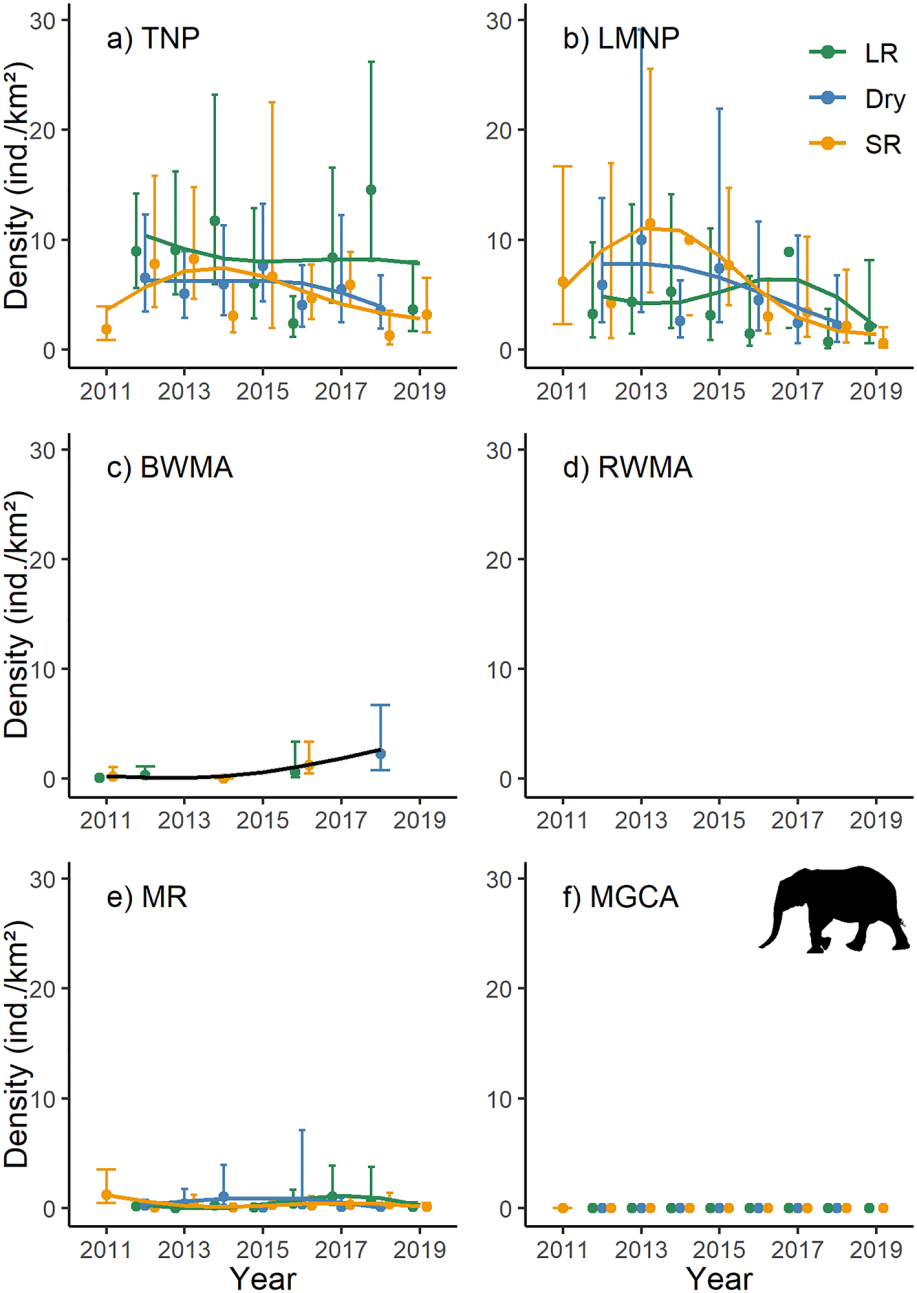
Population density estimates and associated 95% confidence intervals for elephant (*Loxodonta africana*). The trend lines are based on 1000 Monte Carlo replicates and modelled as season-specific (LR: long rains; Dry: dry season; SR: short rains) generalized additive models across six management units (TNP: Tarangire National Park; LMNP: Lake Manyara National Park; BWMA: Burunge Wildlife Management Area; RWMA: Randilen Wildlife Management Area; MR: Manyara Ranch; MGCA: Mto wa Mbu Game Controlled Area) of the Tarangire Ecosystem, northern Tanzania. Population density estimates are based on terrestrial line distance sampling surveys. In RWMA, elephant were not counted during the surveys.

Based on estimates from terrestrial line distance surveys and generalized additive models, elephant densities in Burunge WMA (Fig. 2c) increased over the survey period, fluctuated widely but appeared to remain fairly constant in Tarangire NP (Fig. 2a) and Manyara Ranch (Fig. 2e), and seemed to decline in Lake Manyara NP (Fig. 2b, Table S1). During 24 seasonal surveys, we did not detect any elephant in the Mto wa Mbu GCA (Fig. 2f).

**Figure 3:**
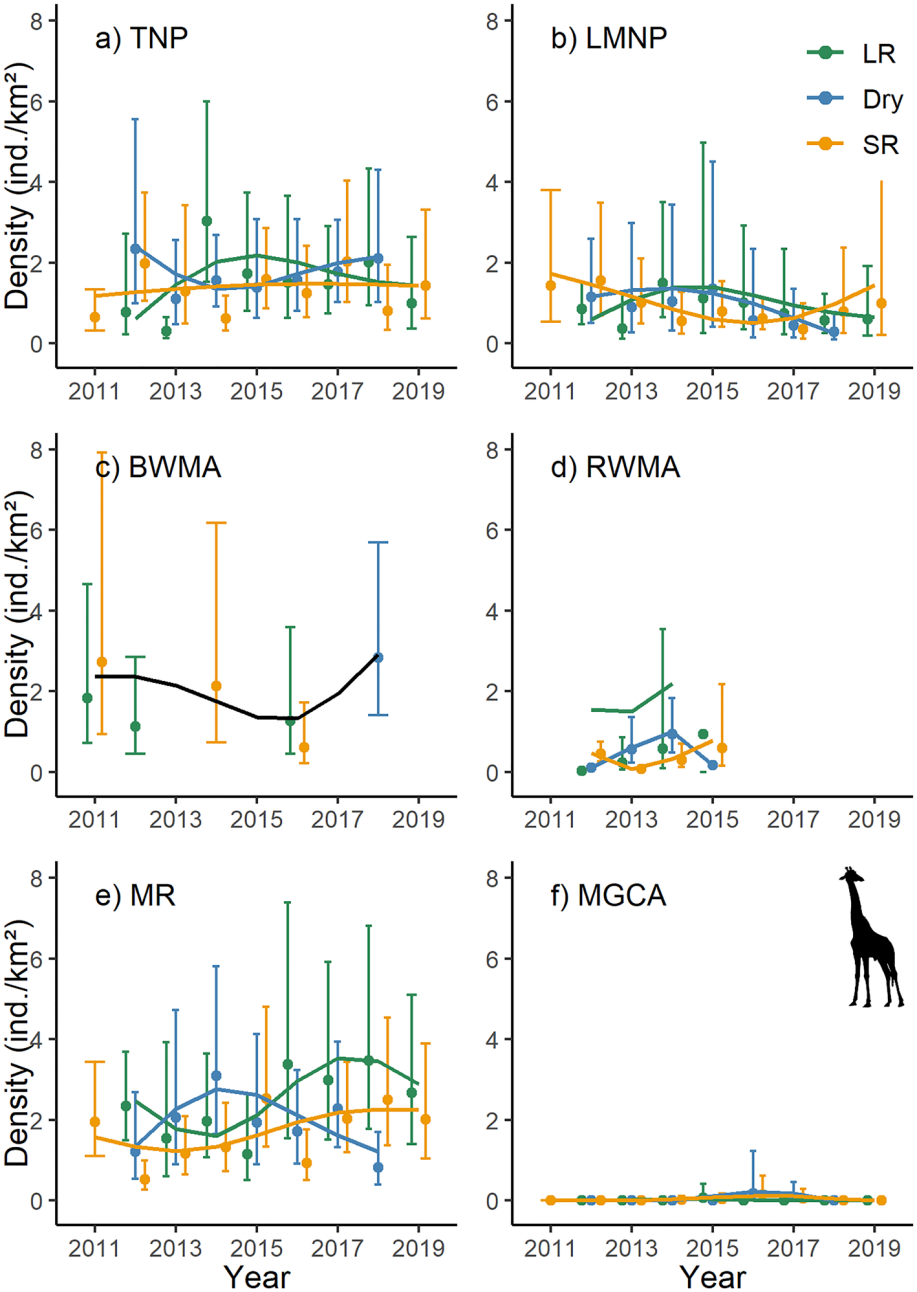
Population density estimates and associated 95% confidence intervals for giraffe (*Giraffa tippelskirchi*). The trend lines are based on 1000 Monte Carlo replicates and modelled as season-specific (LR: long rains; Dry: dry season; SR: short rains) general additive models across six management units (TNP: Tarangire National Park; LMNP: Lake Manyara National Park; BWMA: Burunge Wildlife Management Area; RWMA: Randilen Wildlife Management Area; MR: Manyara Ranch; MGCA: Mto wa Mbu Game Controlled Area) of the Tarangire Ecosystem, northern Tanzania. Population density estimates are based on terrestrial line distance sampling surveys.

Across the monitoring period, giraffe densities showed a slight increase in Tarangire NP (Fig. 3a) and remained relatively constant in both Burunge WMA (Fig. 3c) and Lake Manyara NP (Fig. 3b; Table S1). In Randilen WMA (Fig. 3d) and Manyara Ranch (Fig. 3e), giraffe densities showed a slight upward trend. In the Mto wa Mbu GCA, giraffe densities were much lower than in the other management units (Fig. 3f).

**Figure 4:**
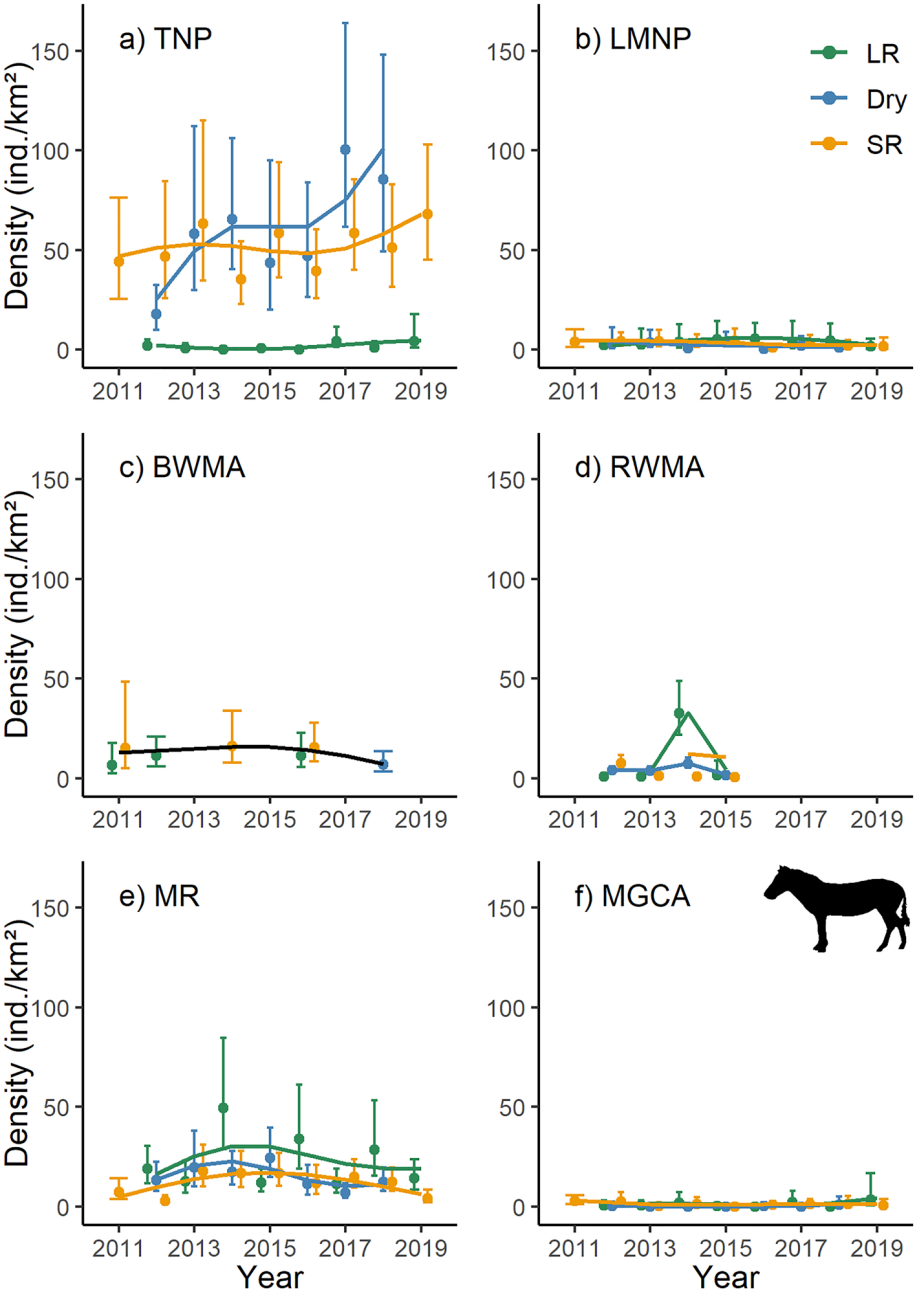
Population density estimates and associated 95% confidence intervals for zebra (*Equus quagga*). The trend lines are based on 1000 Monte Carlo replicates and modelled as season-specific (LR: long rains; Dry: dry season; SR: short rains) generalized additive models across six management units (TNP: Tarangire National Park; LMNP: Lake Manyara National Park; BWMA: Burunge Wildlife Management Area; RWMA: Randilen Wildlife Management Area; MR: Manyara Ranch; MGCA: Mto wa Mbu Game Controlled Area) of the Tarangire Ecosystem, northern Tanzania. Population density estimates are based on terrestrial line distance sampling surveys.

Zebra densities increased markedly in Tarangire NP (Fig. 4a), and remained fairly constant in Lake Manyara NP (Fig. 4b), Burunge WMA (Fig. 4c), Randilen WMA (Fig. 4d), and Manyara Ranch (Fig. 4e; Table S1). In the Mto wa Mbu GCA (Fig. 4f), zebra densities were considerably lower compared to the other management units. Seasonality strongly affected zebra densities in Tarangire NP (Fig. 4a), reflecting their seasonal long-distance movements (i.e. concentration during the dry season inside Tarangire NP, migration to areas outside the NP during the long rains).

**Figure 5:**
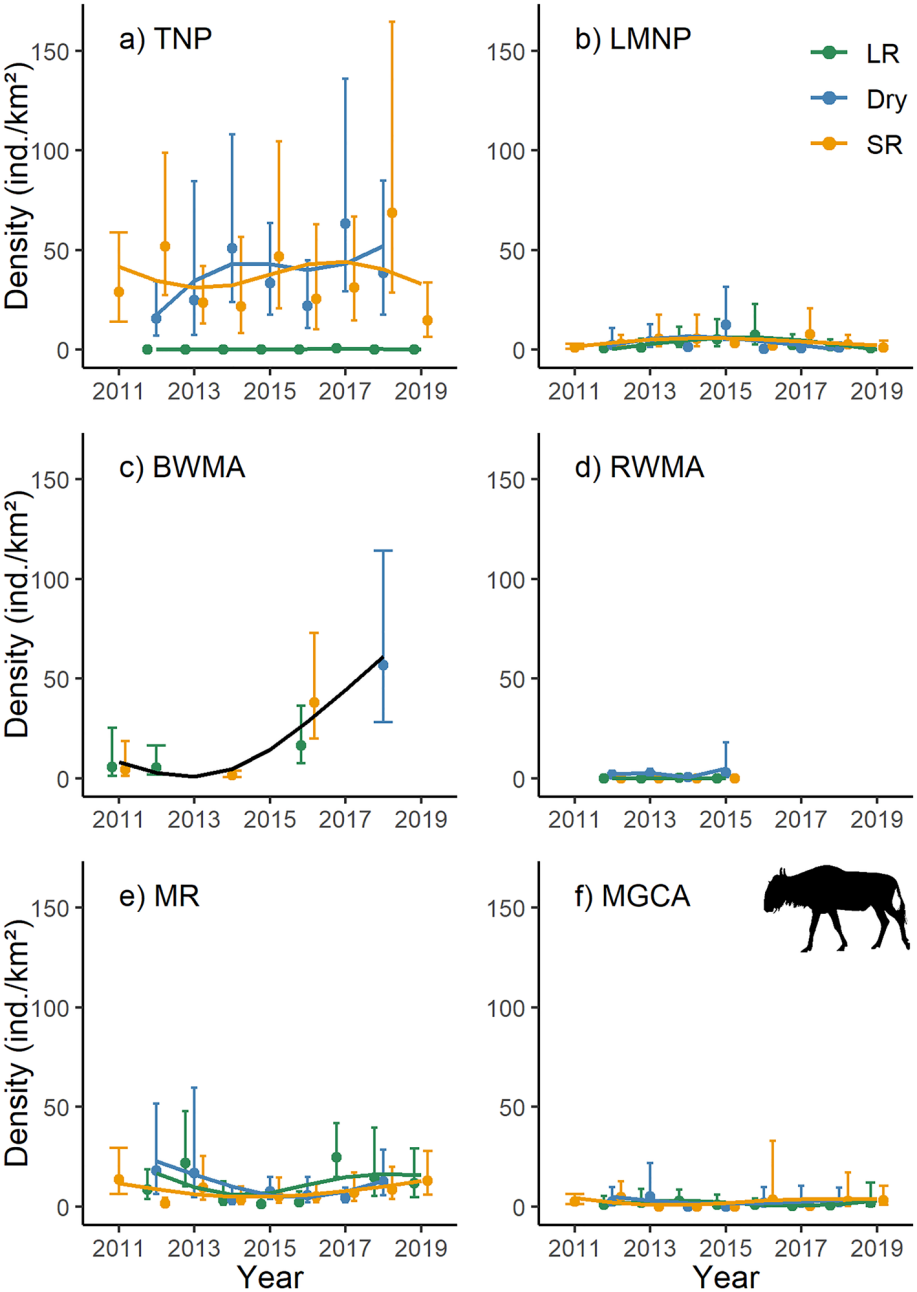
Population density estimates and associated 95% confidence intervals for wildebeest (*Connochaetes taurinus*). The trend lines are based on 1000 Monte Carlo replicates and modelled as season-specific (LR: long rains; Dry: dry season; SR: short rains) generalized additive models across six management units (TNP: Tarangire National Park; LMNP: Lake Manyara National Park; BWMA: Burunge Wildlife Management Area; RWMA: Randilen Wildlife Management Area; MR: Manyara Ranch; MGCA: Mto wa Mbu Game Controlled Area) of the Tarangire Ecosystem, northern Tanzania. Population density estimates are based on terrestrial line distance sampling surveys.

Across the survey period, wildebeest densities remained relatively constant in Tarangire NP (Fig. 5a), with potential increases during the dry season in this core dry season range. In Lake Manyara NP (Fig. 5b) and Manyara Ranch (Fig. 5e; Table S1), their densities did not change substantially over the study period. In Burunge WMA, however, their densities increased markedly (Fig. 5c). Similar to zebra, wildebeest densities were comparably low in Randilen WMA (Fig. 5d) and the Mto wa Mbu GCA (Fig. 5f). As with zebra, seasonality strongly affected wildebeest densities in Tarangire NP, with both species practically absent from the park during the long rains and reaching high densities during the dry season.

## Discussion

**Figure 6:**
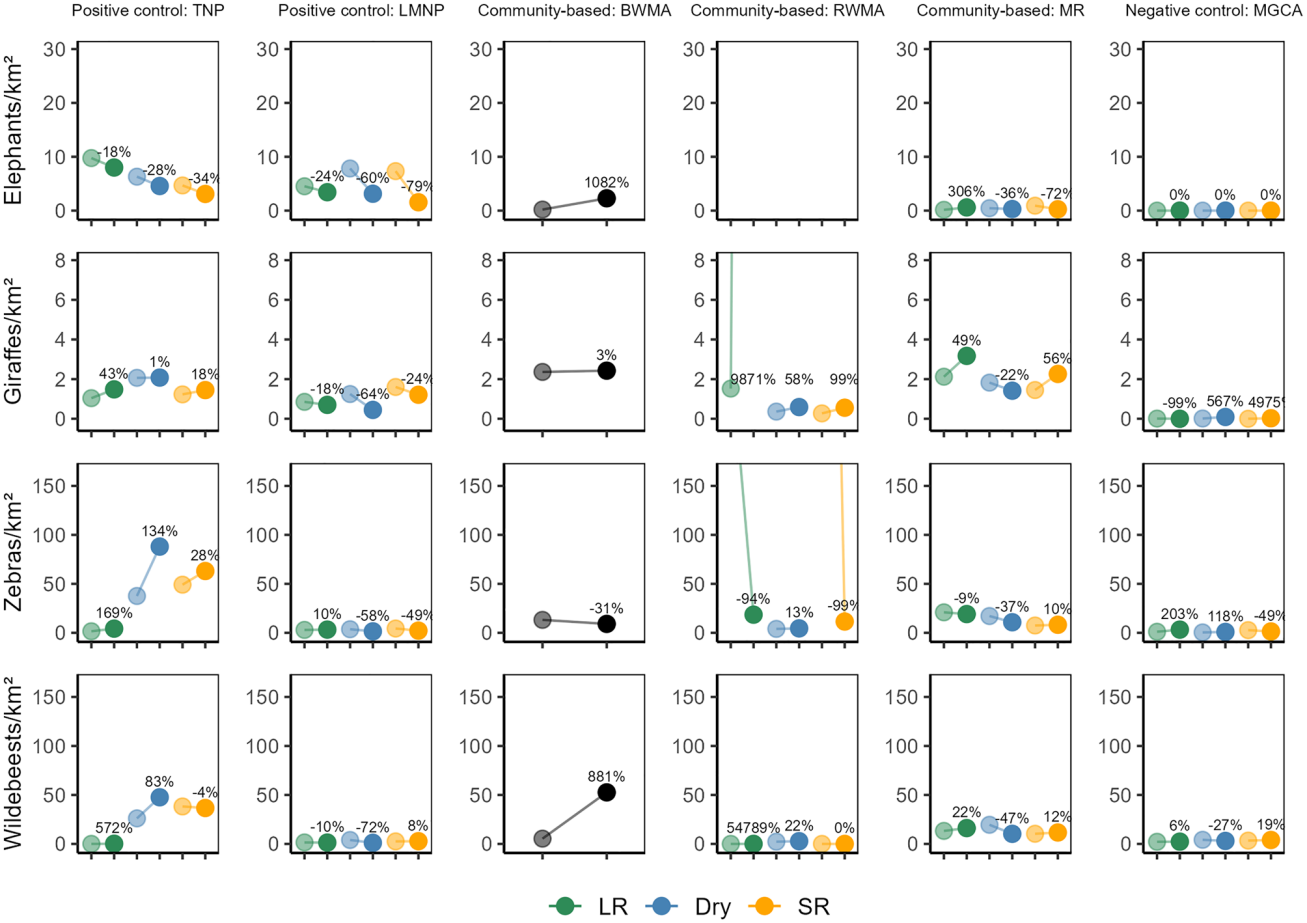
Trends in population density estimates for elephant (*Loxodonta africana*), giraffe (*Giraffa tippelskirchi*), zebra (*Equus quagga*) and wildebeest (*Connochaetes taurinus*) across six management units (TNP: Tarangire National Park; LMNP: Lake Manyara National Park; BWMA: Burunge Wildlife Management Area; RWMA: Randilen Wildlife Management Area; MR: Manyara Ranch; MGCA: Mto wa Mbu Game Controlled Area) in the Tarangire Ecosystem, northern Tanzania. The plotted values represent density estimates for each season (LR: long rains; Dry: dry season; SR: short rains) over different years, based on model predictions. Initial density estimates (lighter circles) are averages from the first two years of the time series, and final density estimates (bolder circles) are from the last two years (note varying time series lengths across areas). Density estimates were derived from terrestrial line distance sampling surveys, using Monte Carlo simulations and generalized additive models to model trends. Percent changes indicate the relative difference in average population density between initial and final periods.

Based on long-term, systematic wildlife monitoring data, we describe population dynamics of four wide-ranging large herbivore species for five protected areas, including three CBCMs, two national parks, and a lesser protected Game Controlled Area in the Tarangire Ecosystem of Tanzania. While our site-based monitoring highlights heterogeneity and seasonality in species-specific densities, we show that densities of the four target species in CBCMs are comparable to those in adjacent Tarangire NP^22^, and occasionally even higher than those in Lake Manyara NP (Fig. 6). Moreover, large herbivore densities were consistently greater than in the Game Controlled Area, which served as a negative control as there are limited conservation efforts in place. Moreover, in Burunge WMA, we detected marked increases in wildebeest and elephant densities over our study period. In Randilen WMA, giraffe densities have increased (Fig. 6). Especially for the central part of the ecosystem (Tarangire NP, Burunge WMA, and Manyara Ranch), population trends of the target species were mostly either stable or indicated population growth over time. This mirrors data from photographic mark-recapture studies of both wildebeest, showing that their population in the ecosystem has stabilized since the early 2000s^34^, as well as giraffe, whose populations in Manyara Ranch and much of Tarangire NP were stable from 2012–2016^37^. Our population trend estimates also align with results of a photographic mark-recapture study conducted from 2012 to 2016, indicating a slight decrease in the giraffe population over that time frame in Burunge WMA^37^ (Fig. 2c); our more recent data suggest that this trajectory has since reversed (Fig. 3c). Overall, our site-based, seasonal monitoring efforts suggest that CBCMs contributed to the stabilization of large herbivore populations, with some areas experiencing increases in numbers, highlighting that strategically placed^28,38^ and locally supported^39^ conservation approaches effectively improved the resilience of wide-ranging herbivore populations in an increasingly fragmented ecosystem.

Our results confirm previous observations that the oldest CBCM model in the ecosystem, Manyara Ranch (established in 2001), sustains densities of giraffe, zebra, and wildebeest that are similar to those observed in adjacent Tarangire NP^19^ and much higher than in the adjacent Mto wa Mbu GCA^26^ as well as a relatively small (compared to Tarangire NP) population of mostly male elephant^40^. These relatively high densities of giraffe, zebra, and wildebeest (and other species^19^) have been sustained over a long time span, suggesting that the concept of Manyara Ranch appears to be working for conserving the current densities of large herbivores. Likely, this is attributed to effective anti-poaching efforts and the enforcement of a limited grazing regime. The grazing strategy aims to build up sufficient grass biomass during the rainy season, allowing for dry season grazing of surrounding communities’ livestock. Data associated with these interventions are currently not accessible, yet analyzing such data would be valuable for assessing the effectiveness of these conservation interventions.

Wildlife monitoring efforts in the Burunge WMA (established in 2006) further highlight the contribution of CBCMs in conserving large herbivores in the ecosystem. Marked increases in elephant densities over time (Fig. 2c), increased density of giraffe after an initial decrease (Fig. 3c), zebra densities greater than outside the area dedicated to wildlife^21^, and a growing wildebeest population (Fig. 5c) that reached densities similar to those observed in neighboring, fully protected Tarangire NP^22^ suggest that Burunge WMA has been effective in conserving large herbivores. This is substantiated by a before-after-control-impact study which documented that adjusting management in Burunge MWA improved giraffe survival^21^.

In the case of the newest CBCM of the ecosystem, Randilen WMA (established in 2014), the contribution to herbivore conservation efforts is perhaps not as obvious. However, as Randilen WMA is not situated in the core migratory routes of the ecosystem^28,41,42^, it is not surprising that zebra and wildebeest densities were low, or occurred sporadically during the rainy season, and did not seem to have increased systematically over the relatively short monitoring period. Nevertheless, the seemingly positive trends in the giraffe population (also supported by more detailed studies within the Randilen WMA^20^) is encouraging and suggest that resident wildlife populations may have benefitted from the implementation of similar management activities to those instituted in Burunge WMA: the effective protection of rangelands from conversion to agriculture or settlements, locally enforced grazing regulations, and community enforcement of anti-poaching measures^20,22^.

During the last two decades, most conservation efforts have focused on areas outside established protected areas in the central part of the ecosystem, i.e. areas around Tarangire NP that are essential for the seasonal migration in and out of the national park. A substantial fraction of these migratory routes are now protected by CBCMs and other community land-use regulations^12,43^, with only a few bottlenecks remaining along the migratory routes^28,42^. Our ground-based monitoring indicates that the zebra population has increased (Fig. 4a) and that the wildebeest population has stabilized and possibly even increased during the dry season (Fig. 5a) in Tarangire NP from 2011 to 2019. As both species spend approximately half of the year outside Tarangire NP in village lands and CBCMs, concentrating in Tarangire NP only during the dry season, these population increases provide circumstantial evidence that conservation measures outside Tarangire NP are effective in bolstering migratory ungulate populations. Since Tarangire NP constitutes the main dry season range for wildebeest and zebra^34,44^, it is likely that these population size trajectories are due to intrinsic population growth and not due to immigration. On the other hand, demographic monitoring of the Tarangire elephant population documented rapid population growth over the past decades, once poaching was effectively curbed in the 1990s^45^. This rapid growth is not evident from our line transect monitoring for the core of Tarangire NP (Fig. 2a).

Our comparison of wildlife trends within the ecosystem (summarized in Fig. 6) supports the idea that conserving functional connectivity^8,13^ is key to supporting populations of large herbivores in the ecosystem. Little to no conservation efforts have been directed towards protecting the remaining connectivity between Lake Manyara NP and the wider ecosystem. While factors internal to Lake Manyara NP (especially bush encroachment)^46^, may have contributed to stagnant and low densities of zebra and wildebeest (which have become largely resident) and a declining population of elephant, it is plausible that the insularization of Lake Manyara NP^47^ contributed to this worrisome development. There remains some connectivity^38^, as evidenced by one documented giraffe movement between Manyara Ranch to Lake Manyara NP and back again^48^. Further, high levels of precipitation in 2019 and 2020 swamped the shortgrass plains habitat along Lake Manyara, and in 2021, no wildebeest were observed in Lake Manyara NP (DEL and MLB, pers. obs.). It is assumed these animals moved out of Lake Manyara NP, again suggesting some connectivity remains.

Similar to Lake Manyara, population densities of resident large herbivores in the Mto wa Mbu GCA, where there are limited anti-poaching measures, are very small (giraffe), functionally absent (elephant), and generally well below historical baselines^35^. Nevertheless, wildlife still occurs in this area and the landscape seems to be permeable for wildlife, especially for wildebeest and zebra which use this area for their annual migration to the Northern Plains^28^, even though agricultural and infrastructural development threaten this functional connectivity in several locations^49^.

Small, isolated populations are more vulnerable to extinction than large, connected populations because of stochastic demographic, environmental, and genetic threats^50^. In light of these threats, and the presence of small populations in the Tarangire Ecosystem such as in Lake Manyara NP, our results suggest that the conservation value of the CBCMs is at least twofold: (1) they effectively increase the area of suitable habitat well beyond core protected areas such as national parks; (2) they support densities of resident large herbivores comparable to those in national parks, thereby increasing effective population sizes of these species in the ecosystem. In addition, CBCMs effectively protect areas that are essential parts of migratory routes in the ecosystem and provide access to seasonally available resources^28,42^, and thus likely contribute to population increases of migratory ungulate populations inside Tarangire NP. Consequently, CBCMs in the Tarangire Ecosystem have been instrumental in preserving space for the annual migration of large herbivores and maintaining ecosystem functioning associated with migratory and resident wildlife populations^51^.

In times of a global biodiversity crisis^52^ and widespread wildlife declines across Africa^2,53^, halting these declines and demonstrating population increases in newly established CBCMs are important steps towards longer-term conservation success. However, such ‘success’ should be viewed within the context of long-term environmental processes and cognitive biases such as the shifting baseline syndrome^54^. We are aware that wildlife populations were historically much more numerous in the Tarangire Ecosystem^31^. Historical anecdotes and long-term data further affirm higher wildlife densities in the Tarangire Ecosystem in the past^31,35^. For instance, based on aerial ecosystem-wide surveys, the wildebeest population in the ecosystem exhibited markedly greater densities during the late 1980s and early 1990s, with an average population size from 1987 to 1994 estimated at approximately 39,000 wildebeest. In contrast, by 2011, it had dwindled to only around 12,000 wildebeest^34^. Because human impacts on wildlife populations started well before the first biodiversity assessments were conducted^55^, we may never know the “true” potential for wildlife densities in the ecosystem, which in any case may have fluctuated considerably. For instance, the first dry season counts of wildebeest and zebra in Tarangire NP during the early 1960s estimated only 1,200 and 2,500 animals, respectively, and while this was likely only a portion of the total Tarangire Ecosystem population, populations of both species had already suffered significant declines in the previous 50 years, which was attributed to a decline in dry season water sources^27^. Given the past and current human population growth rate in Tanzania^56^ and associated need for land required for infrastructure and agriculture^57^, it is debatable if historical wildlife population sizes (if they were ever to be known) are realistic quantitative targets for ecosystem restoration efforts. Critical wildlife habitats, such as dry season concentration areas north of Lake Manyara NP^27^, have been irrevocably lost for wildlife^35^. These substantial and likely irreversible landscape changes have likely reduced the overall carrying capacity for wildlife in the ecosystem. However, it is plausible that wildlife densities in the Tarangire Ecosystem have the potential to increase even under the current extent of core protected areas, combined with the growing extent of multi-use areas where wildlife coexists with livestock and people (e.g. Manyara Ranch, and village lands designated primarily for livestock grazing). Our findings demonstrate that such multi-use, locally managed conservation areas can contribute to the restoration of wildlife numbers in important migratory routes and seasonal ranges, which, with appropriate support, could provide the basis for further wildlife recoveries in the ecosystem over time.

In the last three decades, the main strategy for ecosystem restoration in the Tarangire Ecosystem has been to focus conservation efforts on protecting functional connectivity by creating diverse CBCMs to connect dry and wet season ranges of migratory wildlife. Overall, site-based monitoring suggests that this pragmatic conservation approach was effective. Considering that the majority of wildebeest and zebra spend approximately half of the year outside protected areas, crossing several main roads and traversing areas of human settlement during their seasonal migration, this is a remarkable example for human-wildlife coexistence in a human-dominated landscape, with important implications for conservation policy in East African savanna rangelands and potentially beyond. At the same time, we caution against excessive contentedness with the achieved outcomes. Foremost, several bottlenecks along migratory routes are still threatened by land-use change and expanding settlements; effectively securing these remaining gaps through community-supported actions should be prioritized and may yield a high return on investment for ecosystem conservation efforts^49^. Considering much greater densities of migratory wildlife species during the not too distant past^31^, we encourage conservationists to formulate and strive for bolder goals for the restoration of wildlife populations in the Tarangire Ecosystem. Such ecological restoration goals are likely best achieved if conservation measures are designed as social-ecological endeavors^58^, support Indigenous land and resource tenure and management systems that foster coexistence of livestock and wildlife, ensure that people benefit from increasing wildlife populations, and provide cost-effective ways to minimize costs associated with increasing wildlife populations such as crop damages, livestock depredation, threats to human wellbeing and opportunity, and transaction costs associated with preventing wildlife-related damages. Assessing the effectiveness of such restoration efforts not only requires renewed investment in long-term and ecosystem-wide wildlife monitoring efforts but also in monitoring schemes that assess indicators of social sustainability in CBCMs.

## Methods

### Study area

The climate of the Tarangire Ecosystem is semi-arid and characterized by a bimodal rainfall pattern: a long dry season (June to October) is followed by a short rainy season (November to December), a short dry season (January to February), and then long rains (March to May). Annual precipitation ranges between 434 and 824 mm; the vegetation is characterized by mosaics of *Vachellia-Commiphora* bushland and woodland, edaphic grasslands, and riverine vegetation^59^. The extent of the ecosystem (c. 30,000 km^2^) encompasses the annual movement range of migratory grazers (Fig. 1): during the dry season, zebra and wildebeest mainly concentrate in the northern part of Tarangire NP (total area c. 2650 km^2^) and Manyara Ranch (total area c. 182 km^2^). In these areas, surface water (in Tarangire NP provided by the Tarangire River, the Silale swamps, and some human-enhanced waterholes; in Manyara Ranch provided by the Makuyuni River and human-made dams) and sufficient grass biomass is available during the dry season. At the onset of the short rains, wildebeest and zebra leave Tarangire NP. About half of the wildebeest population migrates eastwards to the Simanjiro Plains and the other half migrates to the northern plains near Lake Natron^44,60^ where females give birth in the nutrient and mineral rich grasslands^61^. Around June, as the surface water on the plains dries up, they return to their dry season ranges.

The eastward migration to the Simanjiro Plains is facilitated by Certificates of Customary Rights of Occupancy (CCRO) that began as conservation easements in 2006. The CCROs secure legal communal title over lands used traditionally for seasonal livestock grazing. These rangelands are conserved by local communities to protect their livestock grazing areas; CCRO by-laws permit livestock keeping but agriculture and settlements are not allowed^12^. The northern migration is strengthened by Manyara Ranch (established in 2001), an unfenced area managed for coexistence between livestock and wildlife; here, pastoralists of two adjacent communities are granted grazing rights and anti-poaching and grazing laws are enforced by rangers^19^. Along the northern migration route, wildlife moves through the Mto wa Mbu GCA, where few restrictions on natural resource extraction are in place^28,35^. Land-use changes from rangeland to agriculture and settlements are constricting wildlife movements^42^. Giraffe^62^ and elephant^63^ do not typically move as far and as predictably as wildebeest and zebra, yet both species have large annual home ranges that exceed the boundaries of protected areas^45,62^. Savanna elephants are mixed feeders (grazing and browsing) and considered a facultative partially migratory species^64^ whereas Masai giraffes are primarily browsers and are considered a resident species with seasonal movements^65^. Both species occur year-round in all of our study sites.

Adjacent to Tarangire NP, several villages established two Wildlife Management Areas, i.e. community-based conservation and development areas that are spatially structured by land-use plans. Burunge WMA (c. 220 km^2^ delineated for wildlife conservation), officially gazetted in 2006, lies to the west of Tarangire NP and connects to Lake Manyara NP^21,22^ whereas Randilen WMA (c. 300 km^2^ delineated for wildlife conservation), established in 2014, is located northeast of Tarangire NP^20^. In the wildlife areas of Burunge and Randilen WMA, agriculture and permanent settlements are not allowed, and village game scouts enforce community land-use regulations and protect wildlife from illegal hunting. The ecosystem also contains the Mkungunero Game Reserve, located between Tarangire NP and the Makame WMA. Some portion of the zebra and wildebeest migrate to these areas during the wet season. However, for these areas we do not have long-term wildlife monitoring data.

Lake Manyara NP (lowland area covers c. 168 km^2^), located at the western edge of the ecosystem, is increasingly isolated due to human development along its northern and southern boundaries. Wildlife in this NP is mostly resident, although occasional movement does occur^42,48^. In Tarangire and Lake Manyara NPs, conservation authorities restrict human use to photographic tourism and research.

### Area-specific density estimates

All wildlife surveys were conducted with permission from the Tanzania Commission for Science and Technology, Tanzania Wildlife Research Institute, Tanzania National Parks, Burunge and Randilen WMA, Manyara Ranch, and the villages of Losirwa, Esilalei, Mswakini, Lolkisale, and Emboret. All methods were carried out in accordance with relevant guidelines and regulations, and our observational studies did not involve experiments with animals. Starting in 2011, we established site-specific wildlife monitoring across multiple management units of the ecosystem. From the end of 2011 to the end of 2019 (see Table 1 for start and end months of the monitoring efforts), we surveyed Lake Manyara NP, Tarangire NP, Manyara Ranch, and the Mto wa Mbu GCA three times per year (24 surveys; in 2019, we omitted the dry season survey) to capture the main seasons^66^. In Burunge WMA, we conducted seven surveys from 2011 to 2018^22^ and in Randilen WMA, we monitored wildlife populations during twelve seasonal surveys from 2012 to 2015^20^. These monitoring efforts were designed as line transect surveys^67^, with transects mostly following roads (except for Burunge WMA, where transects were placed systematically). In Tarangire and Lake Manyara NP, Manyara Ranch, and Mto wa Mbu GCA, transect length was typically 2 km and consecutive transects were separated by 0.5 km; in Burunge WMA, transect length ranged from 0.5 to 10.3 km; in Randilen WMA, we used one single transect per survey (23–85 km, length varied due to road conditions). For most surveys, we used open-top 4 WD vehicles and slowly drove (10–20 km h^-1^) along transects. A minimum of two trained observers counted animals along transects. In Burunge WMA, approximately half of the transects were walked by groups of three persons each. Upon detecting an animal or animal group, we moved to a position perpendicular to the sighting, stopped the vehicle or halted the walk, counted the number of individual animals, and measured the perpendicular distance between the transect and the initial position of the animal using a laser rangefinder. If animals occurred in a group, we measured the perpendicular distance to the approximate center of the group. These data have been analyzed and published previously^19,20,22,46,66^, but we re-analyzed these data for consistency and comparison within the same modeling framework.

To estimate densities we used Distance 6.0^67^. Line distance methodology accounts for imperfect detection and explicitly models the probability $\widehat{P}$ of detecting an animal as a function of the distance from the transect. Due to the non-random placement of transects, density estimates are possibly biased^46^ (but see^19,24^). Therefore, we focus on the temporal trend of density estimates and density comparisons across sites. In cases when surveys were repeated within a single season, we summed effort and sightings^68^. We truncated the farthest 10% of distances, fit species- and area-specific half-normal detection functions with cosine extension^69^, and used the mean cluster size of each season to extrapolate from cluster to animal density. Previously, we tested if detection functions were mediated by season^19,66^; since including this covariate was not supported by model selection, detection functions were pooled across all seasons. Sample size exceeded the recommended threshold of 60 sightings for fitting robust detection functions^68^ in the majority (18/22) of species-area combinations (Table S2). Based on Kolmogorov–Smirnov goodness of fit tests (Table S2), the majority of detection models (16/22) fit the observed data well. Visual assessment of detection functions (Figs. S1–S4) suggested a relatively good fit but also indicated that target species occasionally either avoided (few detections in first distance bins) or were attracted to roads (steep peak of detections in first distance bin). Based on the derived global detection models, we used the post-stratification option in Distance 6.0 for estimating season-specific densities (Table S3). This option allowed us to generate separate density estimates for each season-year-species combination. The density estimate D_ij_ for each area *i* in year-season combination *j* was computed as:

$${D}_{i,j}=\frac{{n}_{ij}}{2w{L}_{ij}\widehat{P}}$$

where ${n}_{ij}$ is the number of detections in stratum* i* during year-season *j*; $w$ is the effective strip half-width; ${L}_{ij}$ is the total length of transects surveyed in stratum *i* during year-season *j*, and $\widehat{P}$ is the global detection function.

### Trend analysis

To assess overall temporal trends for site-specific population trends, we used generalized additive models coupled with a two-stage Monte Carlo sampling approach which enhances the robustness of our analyses by propagating uncertainties from both the distance sampling-derived density estimates and the subsequent trend analysis^53^. Furthermore, this methodological choice was driven by the heterogeneity in our dataset, characterized by unequal year-season combinations across areas. Consequently, we constructed area-, species- and season-specific time series. One exception was the Burunge WMA, where sampling occurred more sporadically. In this case, we aggregated all survey data, disregarding seasonal variations to maintain consistency in trend analysis.

The first stage of our Monte Carlo simulation, implemented in R 4.2.2^70^, involved generating 1000 replicates for each density estimate. We achieved this by simulating data points within the 95% confidence intervals of the original density estimates. For zero densities, we used the normal distribution. For non-zero densities, we employed truncated normal distributions (implemented via the *truncnorm* package^71^), bounded by the lower (*L*) and upper (*U*) limits of the estimated 95% confidence intervals. For each density estimate (*D*), we calculated the standard deviation (*SD*_*estimated*_) as:

$${SD}_{estimated}=\frac{U-L}{2 \times 1.96}$$

We then generated simulated densities (D_simualted_) as follows:

$$\left\{\begin{array}{ll}N \left(0, {SD}_{estimated}\right) & \quad if \; D=0\\ TruncNormal \left(D, {SD}_{estimated}, L, U\right) & \quad if \; D>0\end{array}\right.$$

Subsequently, for each species-, area-, and season-specific time series, we fitted a generalized additive model using the *mgcv* package^72^. In these models, the year (*Y*) was treated as a smooth function, enabling us to capture non-linear temporal trends without presupposing any specific functional form. The target variable for these models was the set of 1,000 simulated data points generated in the first Monte Carlo stage. The model can be expressed as:

$${D}_{simulated}=s(Y, k=4)$$

where $s(Y, k=4)$ represents a smoothing spline function with a basis dimension of 4, allowing for the description of non-linear trends.

The second stage of our Monte Carlo methodology involved using the fitted generalized additive models to predict yearly density estimates. For each predicted year, we generated 1,000 simulated values. These simulations were designed to encompass the uncertainty inherent in the model predictions. We obtained the prediction (D_*predicted*_) and the associated standard error (*SE*) for each year (*Y*_*p*_) from each model. We then simulated new density values based on the predicted mean and standard errors:

$${D}_{simulated\_predictions}\sim N ({\widehat{D}}_{predicted}, SE)$$

We then used the mean values of these 1000 simulated values per year to depict trend lines for each seasonal time series. This two-stage Monte Carlo approach, coupled with the flexibility of generalized additive models, allowed us to produce a nuanced and statistically robust analysis of population dynamics across species and ecological contexts. We visualized the time series by plotting the observed density estimates and the predicted yearly trends in *ggplot2*^73^.

To separate overall population trends from noise (arising from uncertainty in density and trend estimates), we condensed the key information of the time series. For each species, area, and season, we calculated the average density estimates for the initial and final years of the modelled time series. The initial and final density estimates were derived from the first and last two years of the monitoring period, respectively. For Randilen WMA, the initial years were 2012–2013 and the final years were 2014–2015. For Tarangire NP, Lake Manyara NP, Manyara Ranch, and Mto wa Mbu GCA, the initial and final years varied by season: short rains (2011–2012 to 2018–2019), long rains (2012–2013 to 2018–2019), and dry season (2012–2013 to 2017–2018; in 2019 we did not conduct dry season counts). For Burunge WMA, we aggregated data from 2011–2012 and 2017–2018 across all seasons. We then computed the percent change in density between the initial and final periods using the formula:

$$Percent \; change=\left(\frac{Final \; density-Initial \; density}{Initial \;density}\right) \times 100$$

This approach smooths out non-linearity and provides an indication of the direction and magnitude of population changes over the study period.

### Supplementary Information

Supplementary Information.

## Data Availability

Density estimates derived by line distance sampling surveys are available in Table S3. The data and the code for the corresponding trend analyses and Figs. 2, 3, 4, 5, 6 are available at GöttingenResearchOnline: 10.25625/IC16AO.
