# Supplementary material for: The contribution of community-based conservation models to conserving large herbivore populations
Source: Sci Rep. 2024 Jul 13;14:16221. doi: 10.1038/s41598-024-66517-9 (PMC11246445; doi:10.1038/s41598-024-66517-9)

**Table S1.** Parameter estimates of the smooth parameter for the “year “variable and R² values of general additive models which describe annual trends of elephant, giraffe, zebra and wildebeest densities in the Tarangire Ecosystem, northern Tanzania. General additive models were based on season-, area-, and species-specific density estimates derived from line distance surveys. Models were fitted on 1,000 Monte Carlo replicates of seasonal density estimates.

| **Species** | **Area** | **Season** | **Estimated degrees**  **of freedom** | **F-value** | **p-value** | | | **R²** |
| --- | --- | --- | --- | --- | --- | --- | --- | --- |
| Elephant | TNP | SR | 2.997 | 978.846 | | < 0.001 | 24.62% | |
| Elephant | TNP | LR | 2.929 | 67.098 | | < 0.001 | 2.48% | |
| Elephant | TNP | Dry | 2.977 | 474.874 | | < 0.001 | 16.86% | |
| Elephant | LMNP | SR | 2.999 | 3148.563 | | < 0.001 | 51.22% | |
| Elephant | LMNP | LR | 2.992 | 204.005 | | < 0.001 | 7.07% | |
| Elephant | LMNP | Dry | 2.929 | 624.778 | | < 0.001 | 21.13% | |
| Elephant | BWMA | All seasons | 2.995 | 5242.380 | | < 0.001 | 69.20% | |
| Elephant | MR | SR | 2.999 | 2299.965 | | < 0.001 | 43.40% | |
| Elephant | MR | LR | 3.000 | 2665.958 | | < 0.001 | 49.99% | |
| Elephant | MR | Dry | 2.986 | 422.171 | | < 0.001 | 15.30% | |
| Giraffe | TNP | SR | 2.765 | 62.749 | | < 0.001 | 1.99% | |
| Giraffe | TNP | LR | 2.995 | 732.823 | | < 0.001 | 21.58% | |
| Giraffe | TNP | Dry | 2.996 | 717.474 | | < 0.001 | 23.54% | |
| Giraffe | LMNP | SR | 2.998 | 2044.090 | | < 0.001 | 40.51% | |
| Giraffe | LMNP | LR | 2.997 | 814.135 | | < 0.001 | 23.41% | |
| Giraffe | LMNP | Dry | 2.988 | 1266.756 | | < 0.001 | 35.20% | |
| Giraffe | BWMA | All seasons | 2.996 | 636.743 | | < 0.001 | 21.46% | |
| Giraffe | MR | SR | 2.994 | 836.081 | | < 0.001 | 21.80% | |
| Giraffe | MR | LR | 2.999 | 1535.436 | | < 0.001 | 36.54% | |
| Giraffe | MR | Dry | 2.997 | 1316.223 | | < 0.001 | 36.09% | |
| Giraffe | MGCA | SR | 2.999 | 2576.820 | | < 0.001 | 46.20% | |
| Giraffe | MGCA | LR | 2.996 | 607.684 | | < 0.001 | 18.58% | |
| Giraffe | MGCA | Dry | 2.999 | 1909.364 | | < 0.001 | 45.00% | |
| Giraffe | RWMA | SR | 2.997 | 1916.464 | | < 0.001 | 58.96% | |
| Giraffe | RWMA | LR | 2.996 | 1716.883 | | < 0.001 | 56.31% | |
| Giraffe | RWMA | Dry | 2.999 | 5082.285 | | < 0.001 | 79.22% | |
| Zebra | TNP | SR | 2.996 | 549.684 | | < 0.001 | 15.46% | |
| Zebra | TNP | LR | 2.995 | 1775.552 | | < 0.001 | 39.98% | |
| Zebra | TNP | Dry | 2.998 | 2235.406 | | < 0.001 | 48.93% | |
| Zebra | LMNP | SR | 2.989 | 1004.442 | | < 0.001 | 25.09% | |
| Zebra | LMNP | LR | 2.996 | 1142.409 | | < 0.001 | 29.97% | |
| Zebra | LMNP | Dry | 2.882 | 611.691 | | < 0.001 | 20.73% | |
| Zebra | BWMA | All seasons | 2.989 | 494.061 | | < 0.001 | 17.50% | |
| Zebra | MR | SR | 2.998 | 2419.932 | | < 0.001 | 44.65% | |
| Zebra | MR | LR | 2.994 | 313.365 | | < 0.001 | 10.54% | |
| Zebra | MR | Dry | 2.999 | 1844.522 | | < 0.001 | 44.16% | |
| Zebra | MGCA | SR | 2.998 | 1743.075 | | < 0.001 | 36.76% | |
| Zebra | MGCA | LR | 2.998 | 1290.601 | | < 0.001 | 32.60% | |
| Zebra | MGCA | Dry | 2.995 | 1520.831 | | < 0.001 | 39.46% | |
| Zebra | RWMA | SR | 2.999 | 1812.778 | | < 0.001 | 57.61% | |
| Zebra | RWMA | LR | 2.997 | 1681.989 | | < 0.001 | 55.76% | |
| Zebra | RWMA | Dry | 3.000 | 8701.502 | | < 0.001 | 86.72% | |
| Wildebeest | TNP | SR | 2.994 | 138.322 | | < 0.001 | 4.40% | |
| Wildebeest | TNP | LR | 2.999 | 879.468 | | < 0.001 | 24.79% | |
| Wildebeest | TNP | Dry | 2.993 | 597.999 | | < 0.001 | 20.41% | |
| Wildebeest | LMNP | SR | 2.993 | 648.361 | | < 0.001 | 17.79% | |
| Wildebeest | LMNP | LR | 2.998 | 2394.575 | | < 0.001 | 47.31% | |
| Wildebeest | LMNP | Dry | 2.990 | 564.236 | | < 0.001 | 19.50% | |
| Wildebeest | BWMA | All seasons | 2.997 | 7619.099 | | < 0.001 | 76.55% | |
| Wildebeest | MR | SR | 2.995 | 1151.148 | | < 0.001 | 27.74% | |
| Wildebeest | MR | LR | 2.997 | 522.160 | | < 0.001 | 16.39% | |
| Wildebeest | MR | Dry | 2.995 | 1841.158 | | < 0.001 | 44.09% | |
| Wildebeest | MGCA | SR | 2.996 | 396.754 | | < 0.001 | 11.69% | |
| Wildebeest | MGCA | LR | 2.999 | 1403.126 | | < 0.001 | 34.47% | |
| Wildebeest | MGCA | Dry | 2.984 | 420.162 | | < 0.001 | 15.26% | |
| Wildebeest | RWMA | LR | 3.000 | 48209.695 | | < 0.001 | 97.31% | |
| Wildebeest | RWMA | Dry | 2.999 | 1210.291 | | < 0.001 | 47.60% | |

**Table S2.** Key parameters associated with half-normal detection functions with cosine extension used to estimate population densities of wildlife species in different management units (TNP: Tarangire National Park; LMNP: Lake Manyara National Park; BWMA: Burunge Wildlife Management Area, RWMA: Randilen Wildlife Management Area; MR: Manyara Ranch; MGCA: Mto wa Mbu Game Controlled Area) of the Tarangire Ecosystem, northern Tanzania. Detection functions are based on road transect surveys conducted from 2011–2019. ‘n’ indicates the number of detections, “P_a_” the estimated detection probability, including the associated 95%-confidence intervals, “ESW” is the estimated strip width in meters and its associated confidence intervals, and ‘KS p-value’ is the probability of a Kolmogorov-Smirnov goodness-of-fit test.

| **Species** | **Area** | **n** | **P_a_** | **ESW (m)** | **KS P-value** |
| --- | --- | --- | --- | --- | --- |
| Elephant | TNP | 610 | 0.54 (0.49-0.61) | 236 (211-265) | 0.045 |
| Elephant | LMNP | 167 | 0.33 (0.29-0.38) | 44 (39-50) | ≤0.001 |
| Elephant | BWMA | 43 | 0.46 (0.32-0.67) | 170 (118-246) | 0.372 |
| Elephant | RWMA | Not counted |  |  |  |
| Elephant | MR | 66 | 0.65 (0.52-0.81) | 260 (208-324) | 0.938 |
| Elephant | MGCA | 0 |  |  |  |
| Giraffe | TNP | 371 | 0.58 (0.53-0.63) | 199 (183-217) | 0.257 |
| Giraffe | LMNP | 256 | 0.47 (0.41-0.55) | 184 (158-214) | 0.354 |
| Giraffe | BWMA | 94 | 0.80 (0.65-0.97) | 165 (136-200) | 0.817 |
| Giraffe | RWMA | 58 | 0.80 (0.62-1.00) | 285 (221-369) | 0.768 |
| Giraffe | MR | 520 | 0.65 (0.60-0.70) | 238 (221-257) | 0.261 |
| Giraffe | MGCA | 13 | 0.57 (0.38-0.87) | 429 (282-652) | 0.753 |
| Zebra | TNP | 903 | 0.48 (0.45-0.53) | 124 (114-134) | ≤0.001 |
| Zebra | LMNP | 295 | 0.63 (0.54-0.75) | 286 (241-338) | 0.510 |
| Zebra | BWMA | 226 | 0.63 (0.57-0.71) | 185 (165-206) | 0.455 |
| Zebra | RWMA | 151 | 0.75 (0.64-0.87) | 227 (195-263) | 0.613 |
| Zebra | MR | 1147 | 0.61 (0.59-0.65) | 193 (184-203) | 0.001 |
| Zebra | MGCA | 141 | 0.64 (0.55-0.74) | 350 (301-406) | 0.098 |
| Wildebeest | TNP | 435 | 0.60 (0.55-0.65) | 169 (155-183) | 0.093 |
| Wildebeest | LMNP | 299 | 0.81 (0.72-0.91) | 387 (345-436) | 0.068 |
| Wildebeest | BWMA | 148 | 0.68 (0.58-0.79) | 204 (176-238) | 0.040 |
| Wildebeest | RWMA | 29 | 0.65 (0.47-0.90) | 225 (162-312) | 0.979 |
| Wildebeest | MR | 563 | 0.79 (0.65-0.96) | 248 (205-302) | 0.049 |
| Wildebeest | MGCA | 155 | 0.81 (0.69-0.94) | 484 (413-566) | 0.927 |

**Table S3.** Seasonal (LR: long rains; Dry: dry season; SR: short rains) density estimates and associated 95% confidence intervals for elephant, giraffe, zebra, and wildebeest in different management units (TNP: Tarangire National Park; LMNP: Lake Manyara National Park; BWMA: Burunge Wildlife Management Area, RWMA: Randilen Wildlife Management Area; MR: Manyara Ranch; MGCA: Mto wa Mbu Game Controlled Area) of the Tarangire Ecosystem, northern Tanzania.

| Year | Season | Area | Species | Density | lower 95% CI | upper 95% CI |
| --- | --- | --- | --- | --- | --- | --- |
| 2011 | SR | TNP | Elephant | 1.853 | 0.874 | 3.929 |
| 2012 | LR | TNP | Elephant | 8.926 | 5.606 | 14.211 |
| 2012 | Dry | TNP | Elephant | 6.510 | 3.454 | 12.271 |
| 2012 | SR | TNP | Elephant | 7.779 | 3.833 | 15.787 |
| 2013 | LR | TNP | Elephant | 9.028 | 5.023 | 16.228 |
| 2013 | Dry | TNP | Elephant | 5.062 | 2.871 | 8.926 |
| 2013 | SR | TNP | Elephant | 8.232 | 4.587 | 14.771 |
| 2014 | LR | TNP | Elephant | 11.705 | 5.909 | 23.184 |
| 2014 | Dry | TNP | Elephant | 5.951 | 3.133 | 11.303 |
| 2014 | SR | TNP | Elephant | 3.073 | 1.531 | 6.169 |
| 2015 | LR | TNP | Elephant | 6.022 | 2.820 | 12.861 |
| 2015 | Dry | TNP | Elephant | 7.634 | 4.399 | 13.248 |
| 2015 | SR | TNP | Elephant | 6.636 | 1.955 | 22.529 |
| 2016 | LR | TNP | Elephant | 2.340 | 1.126 | 4.863 |
| 2016 | Dry | TNP | Elephant | 4.005 | 2.092 | 7.665 |
| 2016 | SR | TNP | Elephant | 4.649 | 2.787 | 7.754 |
| 2017 | LR | TNP | Elephant | 8.374 | 4.235 | 16.558 |
| 2017 | Dry | TNP | Elephant | 5.460 | 2.443 | 12.204 |
| 2017 | SR | TNP | Elephant | 5.881 | 3.901 | 8.864 |
| 2018 | Dry | TNP | Elephant | 3.576 | 1.893 | 6.757 |
| 2018 | LR | TNP | Elephant | 14.516 | 8.047 | 26.189 |
| 2018 | SR | TNP | Elephant | 1.276 | 0.467 | 3.488 |
| 2019 | LR | TNP | Elephant | 3.640 | 1.659 | 7.988 |
| 2019 | SR | TNP | Elephant | 3.174 | 1.548 | 6.507 |
| 2011 | SR | LMNP | Elephant | 6.148 | 2.270 | 16.654 |
| 2012 | LR | LMNP | Elephant | 3.230 | 1.073 | 9.728 |
| 2012 | Dry | LMNP | Elephant | 5.857 | 2.491 | 13.776 |
| 2012 | SR | LMNP | Elephant | 4.179 | 1.028 | 16.992 |
| 2013 | LR | LMNP | Elephant | 4.315 | 1.407 | 13.234 |
| 2013 | Dry | LMNP | Elephant | 9.953 | 3.398 | 29.157 |
| 2013 | SR | LMNP | Elephant | 11.484 | 5.162 | 25.550 |
| 2014 | LR | LMNP | Elephant | 5.253 | 1.948 | 14.165 |
| 2014 | Dry | LMNP | Elephant | 2.588 | 1.065 | 6.288 |
| 2014 | SR | LMNP | Elephant | 9.976 | 3.125 | 31.851 |
| 2015 | LR | LMNP | Elephant | 3.112 | 0.881 | 10.994 |
| 2015 | Dry | LMNP | Elephant | 7.374 | 2.478 | 21.945 |
| 2015 | SR | LMNP | Elephant | 7.689 | 4.024 | 14.694 |
| 2016 | LR | LMNP | Elephant | 1.456 | 0.318 | 6.667 |
| 2016 | Dry | LMNP | Elephant | 4.474 | 1.715 | 11.675 |
| 2016 | SR | LMNP | Elephant | 3.016 | 1.404 | 6.477 |
| 2017 | LR | LMNP | Elephant | 8.868 | 1.966 | 40.001 |
| 2017 | Dry | LMNP | Elephant | 2.398 | 0.554 | 10.383 |
| 2017 | SR | LMNP | Elephant | 3.392 | 1.123 | 10.243 |
| 2018 | Dry | LMNP | Elephant | 2.187 | 0.708 | 6.756 |
| 2018 | LR | LMNP | Elephant | 0.681 | 0.125 | 3.701 |
| 2018 | SR | LMNP | Elephant | 2.124 | 0.622 | 7.250 |
| 2019 | LR | LMNP | Elephant | 2.094 | 0.540 | 8.112 |
| 2019 | SR | LMNP | Elephant | 0.553 | 0.152 | 2.005 |
| 2011 | LR | BWMA | Elephant | 0.047 | 0.008 | 0.273 |
| 2011 | SR | BWMA | Elephant | 0.184 | 0.032 | 1.058 |
| 2012 | LR | BWMA | Elephant | 0.301 | 0.082 | 1.102 |
| 2014 | SR | BWMA | Elephant | 0.025 | 0.004 | 0.142 |
| 2016 | LR | BWMA | Elephant | 0.614 | 0.113 | 3.338 |
| 2016 | SR | BWMA | Elephant | 1.249 | 0.464 | 3.368 |
| 2018 | Dry | BWMA | Elephant | 2.292 | 0.783 | 6.715 |
| 2012 | Dry | RWMA | Elephant | Not counted |  |  |
| 2012 | LR | RWMA | Elephant | Not counted |  |  |
| 2012 | SR | RWMA | Elephant | Not counted |  |  |
| 2013 | Dry | RWMA | Elephant | Not counted |  |  |
| 2013 | LR | RWMA | Elephant | Not counted |  |  |
| 2013 | SR | RWMA | Elephant | Not counted |  |  |
| 2014 | Dry | RWMA | Elephant | Not counted |  |  |
| 2014 | LR | RWMA | Elephant | Not counted |  |  |
| 2014 | SR | RWMA | Elephant | Not counted |  |  |
| 2015 | Dry | RWMA | Elephant | Not counted |  |  |
| 2015 | LR | RWMA | Elephant | Not counted |  |  |
| 2015 | SR | RWMA | Elephant | Not counted |  |  |
| 2011 | SR | MR | Elephant | 1.220 | 0.425 | 3.499 |
| 2012 | LR | MR | Elephant | 0.166 | 0.060 | 0.461 |
| 2012 | Dry | MR | Elephant | 0.190 | 0.049 | 0.739 |
| 2012 | SR | MR | Elephant | 0.029 | 0.005 | 0.158 |
| 2013 | LR | MR | Elephant | 0.000 | 0.000 | 0.000 |
| 2013 | Dry | MR | Elephant | 0.406 | 0.098 | 1.691 |
| 2013 | SR | MR | Elephant | 0.414 | 0.143 | 1.196 |
| 2014 | LR | MR | Elephant | 0.184 | 0.050 | 0.677 |
| 2014 | Dry | MR | Elephant | 1.002 | 0.256 | 3.918 |
| 2014 | SR | MR | Elephant | 0.068 | 0.013 | 0.365 |
| 2015 | LR | MR | Elephant | 0.025 | 0.005 | 0.134 |
| 2015 | Dry | MR | Elephant | 0.025 | 0.005 | 0.134 |
| 2015 | SR | MR | Elephant | 0.278 | 0.092 | 0.844 |
| 2016 | LR | MR | Elephant | 0.404 | 0.099 | 1.645 |
| 2016 | Dry | MR | Elephant | 0.336 | 0.016 | 7.086 |
| 2016 | SR | MR | Elephant | 0.200 | 0.037 | 1.086 |
| 2017 | LR | MR | Elephant | 1.012 | 0.266 | 3.856 |
| 2017 | Dry | MR | Elephant | 0.123 | 0.017 | 0.891 |
| 2017 | SR | MR | Elephant | 0.260 | 0.068 | 0.992 |
| 2018 | Dry | MR | Elephant | 0.073 | 0.017 | 0.308 |
| 2018 | LR | MR | Elephant | 0.633 | 0.107 | 3.752 |
| 2018 | SR | MR | Elephant | 0.321 | 0.075 | 1.380 |
| 2019 | LR | MR | Elephant | 0.092 | 0.017 | 0.505 |
| 2019 | SR | MR | Elephant | 0.083 | 0.015 | 0.453 |
| 2011 | SR | MGCA | Elephant | 0.000 |  |  |
| 2012 | LR | MGCA | Elephant | 0.000 |  |  |
| 2012 | Dry | MGCA | Elephant | 0.000 |  |  |
| 2012 | SR | MGCA | Elephant | 0.000 |  |  |
| 2013 | LR | MGCA | Elephant | 0.000 |  |  |
| 2013 | Dry | MGCA | Elephant | 0.000 |  |  |
| 2013 | SR | MGCA | Elephant | 0.000 |  |  |
| 2014 | LR | MGCA | Elephant | 0.000 |  |  |
| 2014 | Dry | MGCA | Elephant | 0.000 |  |  |
| 2014 | SR | MGCA | Elephant | 0.000 |  |  |
| 2015 | LR | MGCA | Elephant | 0.000 |  |  |
| 2015 | Dry | MGCA | Elephant | 0.000 |  |  |
| 2015 | SR | MGCA | Elephant | 0.000 |  |  |
| 2016 | LR | MGCA | Elephant | 0.000 |  |  |
| 2016 | Dry | MGCA | Elephant | 0.000 |  |  |
| 2016 | SR | MGCA | Elephant | 0.000 |  |  |
| 2017 | LR | MGCA | Elephant | 0.000 |  |  |
| 2017 | Dry | MGCA | Elephant | 0.000 |  |  |
| 2017 | SR | MGCA | Elephant | 0.000 |  |  |
| 2018 | Dry | MGCA | Elephant | 0.000 |  |  |
| 2018 | LR | MGCA | Elephant | 0.000 |  |  |
| 2018 | SR | MGCA | Elephant | 0.000 |  |  |
| 2019 | LR | MGCA | Elephant | 0.000 |  |  |
| 2019 | SR | MGCA | Elephant | 0.000 |  |  |
| 2011 | SR | TNP | Giraffe | 0.655 | 0.319 | 1.346 |
| 2012 | LR | TNP | Giraffe | 0.783 | 0.226 | 2.722 |
| 2012 | Dry | TNP | Giraffe | 2.346 | 0.990 | 5.563 |
| 2012 | SR | TNP | Giraffe | 1.991 | 1.059 | 3.742 |
| 2013 | LR | TNP | Giraffe | 0.299 | 0.138 | 0.652 |
| 2013 | Dry | TNP | Giraffe | 1.112 | 0.482 | 2.568 |
| 2013 | SR | TNP | Giraffe | 1.293 | 0.487 | 3.428 |
| 2014 | LR | TNP | Giraffe | 3.033 | 1.534 | 5.997 |
| 2014 | Dry | TNP | Giraffe | 1.567 | 0.912 | 2.691 |
| 2014 | SR | TNP | Giraffe | 0.612 | 0.314 | 1.191 |
| 2015 | LR | TNP | Giraffe | 1.736 | 0.804 | 3.745 |
| 2015 | Dry | TNP | Giraffe | 1.389 | 0.628 | 3.074 |
| 2015 | SR | TNP | Giraffe | 1.587 | 0.878 | 2.867 |
| 2016 | LR | TNP | Giraffe | 1.519 | 0.630 | 3.659 |
| 2016 | Dry | TNP | Giraffe | 1.582 | 0.811 | 3.084 |
| 2016 | SR | TNP | Giraffe | 1.254 | 0.648 | 2.426 |
| 2017 | LR | TNP | Giraffe | 1.466 | 0.740 | 2.906 |
| 2017 | Dry | TNP | Giraffe | 1.779 | 1.034 | 3.061 |
| 2017 | SR | TNP | Giraffe | 2.028 | 1.020 | 4.033 |
| 2018 | Dry | TNP | Giraffe | 2.103 | 1.026 | 4.308 |
| 2018 | LR | TNP | Giraffe | 2.020 | 0.942 | 4.331 |
| 2018 | SR | TNP | Giraffe | 0.811 | 0.338 | 1.945 |
| 2019 | LR | TNP | Giraffe | 0.995 | 0.374 | 2.649 |
| 2019 | SR | TNP | Giraffe | 1.433 | 0.618 | 3.322 |
| 2011 | SR | LMNP | Giraffe | 1.436 | 0.542 | 3.804 |
| 2012 | LR | LMNP | Giraffe | 0.860 | 0.483 | 1.531 |
| 2012 | Dry | LMNP | Giraffe | 1.150 | 0.509 | 2.600 |
| 2012 | SR | LMNP | Giraffe | 1.555 | 0.693 | 3.491 |
| 2013 | LR | LMNP | Giraffe | 0.373 | 0.112 | 1.237 |
| 2013 | Dry | LMNP | Giraffe | 0.908 | 0.276 | 2.990 |
| 2013 | SR | LMNP | Giraffe | 1.013 | 0.487 | 2.107 |
| 2014 | LR | LMNP | Giraffe | 1.505 | 0.647 | 3.503 |
| 2014 | Dry | LMNP | Giraffe | 1.048 | 0.319 | 3.446 |
| 2014 | SR | LMNP | Giraffe | 0.559 | 0.245 | 1.272 |
| 2015 | LR | LMNP | Giraffe | 1.120 | 0.252 | 4.971 |
| 2015 | Dry | LMNP | Giraffe | 1.357 | 0.409 | 4.502 |
| 2015 | SR | LMNP | Giraffe | 0.797 | 0.409 | 1.551 |
| 2016 | LR | LMNP | Giraffe | 1.009 | 0.348 | 2.926 |
| 2016 | Dry | LMNP | Giraffe | 0.578 | 0.142 | 2.348 |
| 2016 | SR | LMNP | Giraffe | 0.627 | 0.350 | 1.124 |
| 2017 | LR | LMNP | Giraffe | 0.737 | 0.232 | 2.341 |
| 2017 | Dry | LMNP | Giraffe | 0.452 | 0.151 | 1.352 |
| 2017 | SR | LMNP | Giraffe | 0.349 | 0.122 | 0.995 |
| 2018 | Dry | LMNP | Giraffe | 0.283 | 0.103 | 0.772 |
| 2018 | LR | LMNP | Giraffe | 0.572 | 0.266 | 1.229 |
| 2018 | SR | LMNP | Giraffe | 0.784 | 0.259 | 2.376 |
| 2019 | LR | LMNP | Giraffe | 0.610 | 0.194 | 1.921 |
| 2019 | SR | LMNP | Giraffe | 0.995 | 0.213 | 4.645 |
| 2011 | LR | BWMA | Giraffe | 1.841 | 0.727 | 4.662 |
| 2011 | SR | BWMA | Giraffe | 2.723 | 0.937 | 7.911 |
| 2012 | LR | BWMA | Giraffe | 1.135 | 0.451 | 2.856 |
| 2014 | SR | BWMA | Giraffe | 2.129 | 0.733 | 6.182 |
| 2016 | LR | BWMA | Giraffe | 1.272 | 0.451 | 3.589 |
| 2016 | SR | BWMA | Giraffe | 0.615 | 0.219 | 1.727 |
| 2018 | Dry | BWMA | Giraffe | 2.837 | 1.413 | 5.695 |
| 2012 | Dry | RWMA | Giraffe | 0.106 | 0.059 | 0.191 |
| 2012 | LR | RWMA | Giraffe | 0.031 | 0.024 | 0.040 |
| 2012 | SR | RWMA | Giraffe | 0.453 | 0.272 | 0.757 |
| 2013 | Dry | RWMA | Giraffe | 0.556 | 0.227 | 1.364 |
| 2013 | LR | RWMA | Giraffe | 0.232 | 0.063 | 0.862 |
| 2013 | SR | RWMA | Giraffe | 0.075 | 0.058 | 0.096 |
| 2014 | Dry | RWMA | Giraffe | 0.942 | 0.486 | 1.826 |
| 2014 | LR | RWMA | Giraffe | 0.584 | 0.096 | 3.543 |
| 2014 | SR | RWMA | Giraffe | 0.301 | 0.129 | 0.702 |
| 2015 | Dry | RWMA | Giraffe | 0.173 | 0.134 | 0.224 |
| 2015 | LR | RWMA | Giraffe | 0.936 | 0.001 | 1584.500 |
| 2015 | SR | RWMA | Giraffe | 0.592 | 0.161 | 2.176 |
| 2011 | SR | MR | Giraffe | 1.944 | 1.100 | 3.436 |
| 2012 | LR | MR | Giraffe | 2.350 | 1.493 | 3.698 |
| 2012 | Dry | MR | Giraffe | 1.212 | 0.546 | 2.692 |
| 2012 | SR | MR | Giraffe | 0.518 | 0.271 | 0.987 |
| 2013 | LR | MR | Giraffe | 1.544 | 0.607 | 3.932 |
| 2013 | Dry | MR | Giraffe | 2.053 | 0.893 | 4.720 |
| 2013 | SR | MR | Giraffe | 1.162 | 0.645 | 2.095 |
| 2014 | LR | MR | Giraffe | 1.971 | 1.067 | 3.642 |
| 2014 | Dry | MR | Giraffe | 3.098 | 1.654 | 5.803 |
| 2014 | SR | MR | Giraffe | 1.324 | 0.723 | 2.425 |
| 2015 | LR | MR | Giraffe | 1.156 | 0.506 | 2.640 |
| 2015 | Dry | MR | Giraffe | 1.936 | 0.906 | 4.136 |
| 2015 | SR | MR | Giraffe | 2.535 | 1.337 | 4.808 |
| 2016 | LR | MR | Giraffe | 3.373 | 1.540 | 7.384 |
| 2016 | Dry | MR | Giraffe | 1.716 | 0.908 | 3.240 |
| 2016 | SR | MR | Giraffe | 0.939 | 0.502 | 1.755 |
| 2017 | LR | MR | Giraffe | 2.987 | 1.510 | 5.910 |
| 2017 | Dry | MR | Giraffe | 2.285 | 1.325 | 3.942 |
| 2017 | SR | MR | Giraffe | 2.030 | 1.197 | 3.442 |
| 2018 | Dry | MR | Giraffe | 0.823 | 0.397 | 1.707 |
| 2018 | LR | MR | Giraffe | 3.475 | 1.774 | 6.805 |
| 2018 | SR | MR | Giraffe | 2.500 | 1.376 | 4.543 |
| 2019 | LR | MR | Giraffe | 2.667 | 1.395 | 5.100 |
| 2019 | SR | MR | Giraffe | 2.019 | 1.047 | 3.891 |
| 2011 | SR | MGCA | Giraffe | 0.000 | 0.000 | 0.000 |
| 2012 | LR | MGCA | Giraffe | 0.000 | 0.000 | 0.000 |
| 2012 | Dry | MGCA | Giraffe | 0.000 | 0.000 | 0.000 |
| 2012 | SR | MGCA | Giraffe | 0.000 | 0.000 | 0.000 |
| 2013 | LR | MGCA | Giraffe | 0.000 | 0.000 | 0.000 |
| 2013 | Dry | MGCA | Giraffe | 0.000 | 0.000 | 0.000 |
| 2013 | SR | MGCA | Giraffe | 0.000 | 0.000 | 0.000 |
| 2014 | LR | MGCA | Giraffe | 0.000 | 0.000 | 0.000 |
| 2014 | Dry | MGCA | Giraffe | 0.000 | 0.000 | 0.000 |
| 2014 | SR | MGCA | Giraffe | 0.020 | 0.004 | 0.113 |
| 2015 | LR | MGCA | Giraffe | 0.074 | 0.013 | 0.411 |
| 2015 | Dry | MGCA | Giraffe | 0.000 | 0.000 | 0.000 |
| 2015 | SR | MGCA | Giraffe | 0.045 | 0.011 | 0.183 |
| 2016 | LR | MGCA | Giraffe | 0.000 | 0.000 | 0.000 |
| 2016 | Dry | MGCA | Giraffe | 0.171 | 0.024 | 1.227 |
| 2016 | SR | MGCA | Giraffe | 0.141 | 0.032 | 0.618 |
| 2017 | LR | MGCA | Giraffe | 0.000 | 0.000 | 0.000 |
| 2017 | Dry | MGCA | Giraffe | 0.081 | 0.014 | 0.457 |
| 2017 | SR | MGCA | Giraffe | 0.051 | 0.009 | 0.285 |
| 2018 | Dry | MGCA | Giraffe | 0.000 | 0.000 | 0.000 |
| 2018 | LR | MGCA | Giraffe | 0.000 | 0.000 | 0.000 |
| 2018 | SR | MGCA | Giraffe | 0.000 | 0.000 | 0.000 |
| 2019 | LR | MGCA | Giraffe | 0.000 | 0.000 | 0.000 |
| 2019 | SR | MGCA | Giraffe | 0.000 | 0.000 | 0.000 |
| 2011 | SR | TNP | Zebra | 44.073 | 25.504 | 76.160 |
| 2012 | LR | TNP | Zebra | 2.024 | 0.790 | 5.184 |
| 2012 | Dry | TNP | Zebra | 17.954 | 9.950 | 32.397 |
| 2012 | SR | TNP | Zebra | 46.665 | 25.767 | 84.513 |
| 2013 | LR | TNP | Zebra | 0.571 | 0.107 | 3.055 |
| 2013 | Dry | TNP | Zebra | 57.992 | 29.970 | 112.220 |
| 2013 | SR | TNP | Zebra | 63.052 | 34.567 | 115.010 |
| 2014 | LR | TNP | Zebra | 0.000 | 0.000 | 0.000 |
| 2014 | Dry | TNP | Zebra | 65.456 | 40.435 | 105.960 |
| 2014 | SR | TNP | Zebra | 35.277 | 22.920 | 54.297 |
| 2015 | LR | TNP | Zebra | 0.623 | 0.170 | 2.277 |
| 2015 | Dry | TNP | Zebra | 43.593 | 19.997 | 95.032 |
| 2015 | SR | TNP | Zebra | 58.350 | 36.174 | 94.120 |
| 2016 | LR | TNP | Zebra | 0.000 | 0.000 | 0.000 |
| 2016 | Dry | TNP | Zebra | 47.124 | 26.505 | 83.785 |
| 2016 | SR | TNP | Zebra | 39.410 | 25.717 | 60.393 |
| 2017 | LR | TNP | Zebra | 4.019 | 1.392 | 11.604 |
| 2017 | Dry | TNP | Zebra | 100.470 | 61.605 | 163.850 |
| 2017 | SR | TNP | Zebra | 58.570 | 40.116 | 85.513 |
| 2018 | Dry | TNP | Zebra | 85.389 | 49.280 | 147.960 |
| 2018 | LR | TNP | Zebra | 0.899 | 0.191 | 4.229 |
| 2018 | SR | TNP | Zebra | 51.010 | 31.437 | 82.770 |
| 2019 | LR | TNP | Zebra | 4.107 | 0.949 | 17.769 |
| 2019 | SR | TNP | Zebra | 68.114 | 45.117 | 102.830 |
| 2011 | SR | LMNP | Zebra | 3.769 | 1.404 | 10.122 |
| 2012 | LR | LMNP | Zebra | 2.210 | 1.040 | 4.695 |
| 2012 | Dry | LMNP | Zebra | 2.941 | 0.775 | 11.154 |
| 2012 | SR | LMNP | Zebra | 4.069 | 1.897 | 8.729 |
| 2013 | LR | LMNP | Zebra | 2.566 | 0.630 | 10.446 |
| 2013 | Dry | LMNP | Zebra | 3.606 | 1.296 | 10.030 |
| 2013 | SR | LMNP | Zebra | 4.131 | 1.733 | 9.849 |
| 2014 | LR | LMNP | Zebra | 3.703 | 1.085 | 12.639 |
| 2014 | Dry | LMNP | Zebra | 0.676 | 0.164 | 2.795 |
| 2014 | SR | LMNP | Zebra | 3.115 | 1.269 | 7.645 |
| 2015 | LR | LMNP | Zebra | 5.139 | 1.836 | 14.384 |
| 2015 | Dry | LMNP | Zebra | 3.806 | 1.634 | 8.865 |
| 2015 | SR | LMNP | Zebra | 4.451 | 1.871 | 10.587 |
| 2016 | LR | LMNP | Zebra | 5.560 | 2.315 | 13.350 |
| 2016 | Dry | LMNP | Zebra | 0.293 | 0.098 | 0.881 |
| 2016 | SR | LMNP | Zebra | 1.021 | 0.414 | 2.517 |
| 2017 | LR | LMNP | Zebra | 3.301 | 0.757 | 14.387 |
| 2017 | Dry | LMNP | Zebra | 1.936 | 0.569 | 6.585 |
| 2017 | SR | LMNP | Zebra | 3.151 | 1.344 | 7.387 |
| 2018 | Dry | LMNP | Zebra | 0.990 | 0.297 | 3.293 |
| 2018 | LR | LMNP | Zebra | 4.429 | 1.491 | 13.156 |
| 2018 | SR | LMNP | Zebra | 1.796 | 0.657 | 4.911 |
| 2019 | LR | LMNP | Zebra | 1.575 | 0.456 | 5.435 |
| 2019 | SR | LMNP | Zebra | 1.584 | 0.407 | 6.159 |
| 2011 | LR | BWMA | Zebra | 6.523 | 2.396 | 17.759 |
| 2011 | SR | BWMA | Zebra | 15.224 | 4.779 | 48.501 |
| 2012 | LR | BWMA | Zebra | 11.207 | 6.016 | 20.879 |
| 2014 | SR | BWMA | Zebra | 16.092 | 7.684 | 33.701 |
| 2016 | LR | BWMA | Zebra | 11.230 | 5.543 | 22.754 |
| 2016 | SR | BWMA | Zebra | 15.322 | 8.401 | 27.943 |
| 2018 | Dry | BWMA | Zebra | 6.764 | 3.393 | 13.481 |
| 2012 | Dry | RWMA | Zebra | 4.080 | 2.860 | 5.819 |
| 2012 | LR | RWMA | Zebra | 0.891 | 0.000 | 2700.400 |
| 2012 | SR | RWMA | Zebra | 7.582 | 5.022 | 11.447 |
| 2013 | Dry | RWMA | Zebra | 3.713 | 2.290 | 6.021 |
| 2013 | LR | RWMA | Zebra | 0.766 | 0.561 | 1.045 |
| 2013 | SR | RWMA | Zebra | 1.268 | 0.000 | 10993.000 |
| 2014 | Dry | RWMA | Zebra | 7.311 | 5.230 | 10.220 |
| 2014 | LR | RWMA | Zebra | 32.488 | 21.689 | 48.663 |
| 2014 | SR | RWMA | Zebra | 0.831 | 0.383 | 1.801 |
| 2015 | Dry | RWMA | Zebra | 1.580 | 0.760 | 3.286 |
| 2015 | LR | RWMA | Zebra | 1.598 | 0.287 | 8.886 |
| 2015 | SR | RWMA | Zebra | 0.537 | 0.204 | 1.416 |
| 2011 | SR | MR | Zebra | 7.446 | 3.873 | 14.315 |
| 2012 | LR | MR | Zebra | 18.930 | 11.822 | 30.313 |
| 2012 | Dry | MR | Zebra | 13.388 | 7.931 | 22.600 |
| 2012 | SR | MR | Zebra | 2.807 | 1.380 | 5.706 |
| 2013 | LR | MR | Zebra | 12.701 | 6.988 | 23.084 |
| 2013 | Dry | MR | Zebra | 19.671 | 10.157 | 38.095 |
| 2013 | SR | MR | Zebra | 17.678 | 10.030 | 31.156 |
| 2014 | LR | MR | Zebra | 49.465 | 28.824 | 84.887 |
| 2014 | Dry | MR | Zebra | 17.614 | 11.140 | 27.849 |
| 2014 | SR | MR | Zebra | 16.671 | 9.908 | 28.048 |
| 2015 | LR | MR | Zebra | 11.994 | 7.537 | 19.087 |
| 2015 | Dry | MR | Zebra | 24.420 | 15.016 | 39.713 |
| 2015 | SR | MR | Zebra | 16.755 | 10.380 | 27.043 |
| 2016 | LR | MR | Zebra | 34.071 | 18.942 | 61.281 |
| 2016 | Dry | MR | Zebra | 11.376 | 6.184 | 20.927 |
| 2016 | SR | MR | Zebra | 11.600 | 6.469 | 20.800 |
| 2017 | LR | MR | Zebra | 11.398 | 6.878 | 18.889 |
| 2017 | Dry | MR | Zebra | 7.077 | 4.294 | 11.663 |
| 2017 | SR | MR | Zebra | 15.054 | 9.465 | 23.943 |
| 2018 | Dry | MR | Zebra | 12.498 | 7.897 | 19.781 |
| 2018 | LR | MR | Zebra | 28.692 | 15.471 | 53.211 |
| 2018 | SR | MR | Zebra | 12.393 | 7.829 | 19.618 |
| 2019 | LR | MR | Zebra | 14.379 | 8.708 | 23.744 |
| 2019 | SR | MR | Zebra | 4.282 | 2.128 | 8.616 |
| 2011 | SR | MGCA | Zebra | 2.767 | 1.361 | 5.622 |
| 2012 | LR | MGCA | Zebra | 0.485 | 0.077 | 3.039 |
| 2012 | Dry | MGCA | Zebra | 0.451 | 0.083 | 2.448 |
| 2012 | SR | MGCA | Zebra | 2.548 | 0.895 | 7.252 |
| 2013 | LR | MGCA | Zebra | 0.617 | 0.120 | 3.181 |
| 2013 | Dry | MGCA | Zebra | 0.000 | 0.000 | 0.000 |
| 2013 | SR | MGCA | Zebra | 0.449 | 0.155 | 1.299 |
| 2014 | LR | MGCA | Zebra | 1.786 | 0.429 | 7.432 |
| 2014 | Dry | MGCA | Zebra | 0.000 | 0.000 | 0.000 |
| 2014 | SR | MGCA | Zebra | 1.233 | 0.323 | 4.706 |
| 2015 | LR | MGCA | Zebra | 0.409 | 0.106 | 1.581 |
| 2015 | Dry | MGCA | Zebra | 0.000 | 0.000 | 0.000 |
| 2015 | SR | MGCA | Zebra | 0.143 | 0.038 | 0.543 |
| 2016 | LR | MGCA | Zebra | 0.000 | 0.000 | 0.000 |
| 2016 | Dry | MGCA | Zebra | 0.398 | 0.076 | 2.103 |
| 2016 | SR | MGCA | Zebra | 0.910 | 0.285 | 2.910 |
| 2017 | LR | MGCA | Zebra | 2.193 | 0.610 | 7.884 |
| 2017 | Dry | MGCA | Zebra | 0.000 | 0.000 | 0.000 |
| 2017 | SR | MGCA | Zebra | 1.178 | 0.371 | 3.737 |
| 2018 | Dry | MGCA | Zebra | 0.885 | 0.154 | 5.072 |
| 2018 | LR | MGCA | Zebra | 0.000 | 0.000 | 0.000 |
| 2018 | SR | MGCA | Zebra | 1.341 | 0.324 | 5.543 |
| 2019 | LR | MGCA | Zebra | 3.497 | 0.728 | 16.800 |
| 2019 | SR | MGCA | Zebra | 0.787 | 0.166 | 3.736 |
| 2011 | SR | TNP | Wildebeest | 28.814 | 14.148 | 58.685 |
| 2012 | LR | TNP | Wildebeest | 0.000 | 0.000 | 0.000 |
| 2012 | Dry | TNP | Wildebeest | 15.670 | 7.148 | 34.356 |
| 2012 | SR | TNP | Wildebeest | 51.874 | 27.229 | 98.825 |
| 2013 | LR | TNP | Wildebeest | 0.000 | 0.000 | 0.000 |
| 2013 | Dry | TNP | Wildebeest | 24.763 | 7.261 | 84.449 |
| 2013 | SR | TNP | Wildebeest | 23.409 | 13.042 | 42.019 |
| 2014 | LR | TNP | Wildebeest | 0.000 | 0.000 | 0.000 |
| 2014 | Dry | TNP | Wildebeest | 50.805 | 23.886 | 108.060 |
| 2014 | SR | TNP | Wildebeest | 21.617 | 8.280 | 56.438 |
| 2015 | LR | TNP | Wildebeest | 0.000 | 0.000 | 0.000 |
| 2015 | Dry | TNP | Wildebeest | 33.235 | 17.423 | 63.396 |
| 2015 | SR | TNP | Wildebeest | 46.650 | 20.813 | 104.560 |
| 2016 | LR | TNP | Wildebeest | 0.000 | 0.000 | 0.000 |
| 2016 | Dry | TNP | Wildebeest | 21.968 | 10.768 | 44.816 |
| 2016 | SR | TNP | Wildebeest | 25.490 | 10.331 | 62.894 |
| 2017 | LR | TNP | Wildebeest | 0.525 | 0.125 | 2.196 |
| 2017 | Dry | TNP | Wildebeest | 63.210 | 29.392 | 135.940 |
| 2017 | SR | TNP | Wildebeest | 31.151 | 14.524 | 66.813 |
| 2018 | Dry | TNP | Wildebeest | 38.472 | 17.442 | 84.855 |
| 2018 | LR | TNP | Wildebeest | 0.000 | 0.000 | 0.000 |
| 2018 | SR | TNP | Wildebeest | 68.627 | 28.610 | 164.610 |
| 2019 | LR | TNP | Wildebeest | 0.087 | 0.016 | 0.475 |
| 2019 | SR | TNP | Wildebeest | 14.749 | 6.443 | 33.761 |
| 2011 | SR | LMNP | Wildebeest | 1.015 | 0.343 | 3.001 |
| 2012 | LR | LMNP | Wildebeest | 0.697 | 0.183 | 2.652 |
| 2012 | Dry | LMNP | Wildebeest | 2.370 | 0.512 | 10.978 |
| 2012 | SR | LMNP | Wildebeest | 2.841 | 1.089 | 7.413 |
| 2013 | LR | LMNP | Wildebeest | 1.064 | 0.204 | 5.545 |
| 2013 | Dry | LMNP | Wildebeest | 4.271 | 1.437 | 12.697 |
| 2013 | SR | LMNP | Wildebeest | 5.541 | 1.756 | 17.484 |
| 2014 | LR | LMNP | Wildebeest | 3.705 | 1.212 | 11.325 |
| 2014 | Dry | LMNP | Wildebeest | 1.292 | 0.238 | 7.029 |
| 2014 | SR | LMNP | Wildebeest | 5.446 | 1.699 | 17.458 |
| 2015 | LR | LMNP | Wildebeest | 5.012 | 1.662 | 15.111 |
| 2015 | Dry | LMNP | Wildebeest | 12.427 | 4.914 | 31.423 |
| 2015 | SR | LMNP | Wildebeest | 3.231 | 1.700 | 6.139 |
| 2016 | LR | LMNP | Wildebeest | 7.458 | 2.429 | 22.896 |
| 2016 | Dry | LMNP | Wildebeest | 0.412 | 0.093 | 1.824 |
| 2016 | SR | LMNP | Wildebeest | 1.937 | 0.890 | 4.215 |
| 2017 | LR | LMNP | Wildebeest | 2.374 | 0.752 | 7.496 |
| 2017 | Dry | LMNP | Wildebeest | 0.782 | 0.275 | 2.224 |
| 2017 | SR | LMNP | Wildebeest | 7.742 | 2.887 | 20.766 |
| 2018 | Dry | LMNP | Wildebeest | 0.864 | 0.206 | 3.618 |
| 2018 | LR | LMNP | Wildebeest | 1.529 | 0.457 | 5.113 |
| 2018 | SR | LMNP | Wildebeest | 2.507 | 0.843 | 7.455 |
| 2019 | LR | LMNP | Wildebeest | 0.512 | 0.116 | 2.254 |
| 2019 | SR | LMNP | Wildebeest | 0.915 | 0.191 | 4.388 |
| 2011 | LR | BWMA | Wildebeest | 5.576 | 1.231 | 25.253 |
| 2011 | SR | BWMA | Wildebeest | 4.387 | 1.030 | 18.679 |
| 2012 | LR | BWMA | Wildebeest | 5.142 | 1.618 | 16.346 |
| 2014 | SR | BWMA | Wildebeest | 1.506 | 0.635 | 3.574 |
| 2016 | LR | BWMA | Wildebeest | 16.519 | 7.520 | 36.289 |
| 2016 | SR | BWMA | Wildebeest | 37.987 | 19.807 | 72.852 |
| 2018 | Dry | BWMA | Wildebeest | 56.596 | 28.084 | 114.050 |
| 2012 | Dry | RWMA | Wildebeest | 1.838 | 0.885 | 3.815 |
| 2012 | LR | RWMA | Wildebeest | 0.000 | 0.000 | 0.000 |
| 2012 | SR | RWMA | Wildebeest | 0.000 | 0.000 | 0.000 |
| 2013 | Dry | RWMA | Wildebeest | 2.461 | 1.270 | 4.770 |
| 2013 | LR | RWMA | Wildebeest | 0.000 | 0.000 | 0.000 |
| 2013 | SR | RWMA | Wildebeest | 0.000 | 0.000 | 0.000 |
| 2014 | Dry | RWMA | Wildebeest | 0.556 | 0.226 | 1.370 |
| 2014 | LR | RWMA | Wildebeest | 0.059 | 0.043 | 0.082 |
| 2014 | SR | RWMA | Wildebeest | 0.000 | 0.000 | 0.000 |
| 2015 | Dry | RWMA | Wildebeest | 2.665 | 0.399 | 17.803 |
| 2015 | LR | RWMA | Wildebeest | 0.000 | 0.000 | 0.000 |
| 2015 | SR | RWMA | Wildebeest | 0.000 | 0.000 | 0.000 |
| 2011 | SR | MR | Wildebeest | 13.649 | 6.333 | 29.416 |
| 2012 | LR | MR | Wildebeest | 8.513 | 3.865 | 18.754 |
| 2012 | Dry | MR | Wildebeest | 18.072 | 6.314 | 51.727 |
| 2012 | SR | MR | Wildebeest | 1.429 | 0.466 | 4.383 |
| 2013 | LR | MR | Wildebeest | 21.979 | 10.096 | 47.851 |
| 2013 | Dry | MR | Wildebeest | 16.883 | 4.777 | 59.668 |
| 2013 | SR | MR | Wildebeest | 9.494 | 3.542 | 25.445 |
| 2014 | LR | MR | Wildebeest | 3.263 | 0.843 | 12.630 |
| 2014 | Dry | MR | Wildebeest | 3.354 | 1.141 | 9.865 |
| 2014 | SR | MR | Wildebeest | 3.602 | 1.268 | 10.230 |
| 2015 | LR | MR | Wildebeest | 1.264 | 0.266 | 6.013 |
| 2015 | Dry | MR | Wildebeest | 7.638 | 3.900 | 14.957 |
| 2015 | SR | MR | Wildebeest | 5.157 | 1.816 | 14.645 |
| 2016 | LR | MR | Wildebeest | 2.064 | 0.569 | 7.491 |
| 2016 | Dry | MR | Wildebeest | 5.724 | 2.198 | 14.911 |
| 2016 | SR | MR | Wildebeest | 5.054 | 2.235 | 11.425 |
| 2017 | LR | MR | Wildebeest | 24.914 | 14.860 | 41.772 |
| 2017 | Dry | MR | Wildebeest | 4.464 | 2.082 | 9.570 |
| 2017 | SR | MR | Wildebeest | 7.029 | 2.904 | 17.015 |
| 2018 | Dry | MR | Wildebeest | 12.815 | 5.768 | 28.469 |
| 2018 | LR | MR | Wildebeest | 14.491 | 5.313 | 39.523 |
| 2018 | SR | MR | Wildebeest | 8.644 | 3.729 | 20.040 |
| 2019 | LR | MR | Wildebeest | 11.839 | 4.779 | 29.331 |
| 2019 | SR | MR | Wildebeest | 12.868 | 5.939 | 27.883 |
| 2011 | SR | MGCA | Wildebeest | 2.633 | 1.114 | 6.224 |
| 2012 | LR | MGCA | Wildebeest | 1.005 | 0.192 | 5.267 |
| 2012 | Dry | MGCA | Wildebeest | 2.607 | 0.683 | 9.950 |
| 2012 | SR | MGCA | Wildebeest | 4.682 | 1.719 | 12.753 |
| 2013 | LR | MGCA | Wildebeest | 1.869 | 0.394 | 8.868 |
| 2013 | Dry | MGCA | Wildebeest | 5.153 | 1.205 | 22.039 |
| 2013 | SR | MGCA | Wildebeest | 0.000 | 0.000 | 0.000 |
| 2014 | LR | MGCA | Wildebeest | 2.771 | 0.895 | 8.578 |
| 2014 | Dry | MGCA | Wildebeest | 0.000 | 0.000 | 0.000 |
| 2014 | SR | MGCA | Wildebeest | 0.000 | 0.000 | 0.000 |
| 2015 | LR | MGCA | Wildebeest | 1.034 | 0.180 | 5.954 |
| 2015 | Dry | MGCA | Wildebeest | 0.000 | 0.000 | 0.000 |
| 2015 | SR | MGCA | Wildebeest | 0.000 | 0.000 | 0.000 |
| 2016 | LR | MGCA | Wildebeest | 1.005 | 0.241 | 4.190 |
| 2016 | Dry | MGCA | Wildebeest | 1.849 | 0.343 | 9.974 |
| 2016 | SR | MGCA | Wildebeest | 3.446 | 0.361 | 32.855 |
| 2017 | LR | MGCA | Wildebeest | 0.310 | 0.039 | 2.466 |
| 2017 | Dry | MGCA | Wildebeest | 1.924 | 0.351 | 10.540 |
| 2017 | SR | MGCA | Wildebeest | 0.167 | 0.046 | 0.609 |
| 2018 | Dry | MGCA | Wildebeest | 2.260 | 0.533 | 9.584 |
| 2018 | LR | MGCA | Wildebeest | 0.711 | 0.122 | 4.138 |
| 2018 | SR | MGCA | Wildebeest | 2.714 | 0.429 | 17.179 |
| 2019 | LR | MGCA | Wildebeest | 1.761 | 0.258 | 12.027 |
| 2019 | SR | MGCA | Wildebeest | 3.325 | 1.042 | 10.618 |

**Fig S1.** Detection functions of elephants in different management units (TNP: Tarangire National Park; LMNP: Lake Manyara National Park; BWMA: Burunge Wildlife Management Area, RWMA: Randilen Wildlife Management Area; MR: Manyara Ranch; MGCA: Mto wa Mbu Game Controlled Area) of the Tarangire Ecosystem, northern Tanzania.


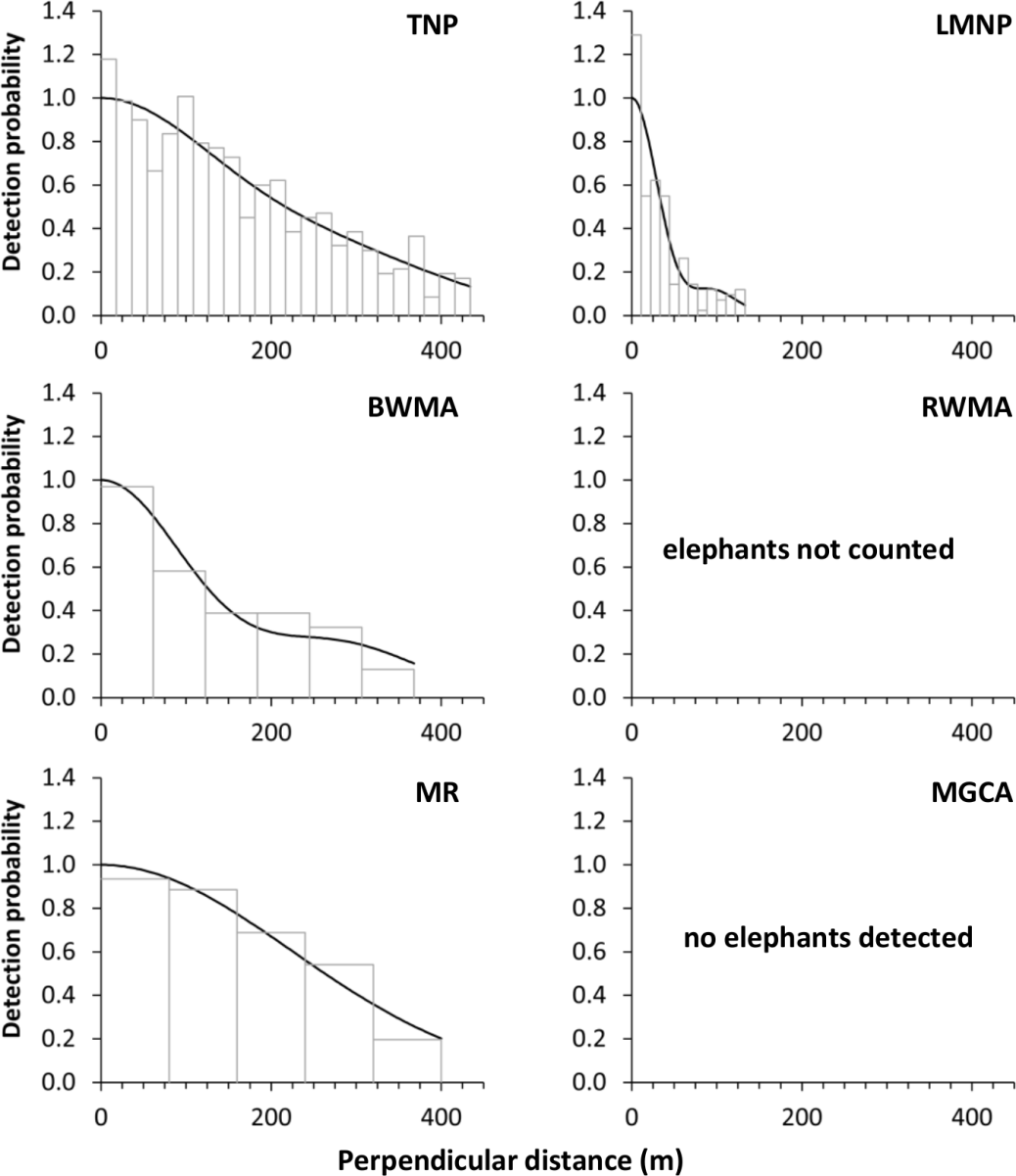


**Fig S2.** Detection functions of giraffes in different management units (TNP: Tarangire National Park; LMNP: Lake Manyara National Park; BWMA: Burunge Wildlife Management Area, RWMA: Randilen Wildlife Management Area; MR: Manyara Ranch; MGCA: Mto wa Mbu Game Controlled Area) of the Tarangire Ecosystem, northern Tanzania.


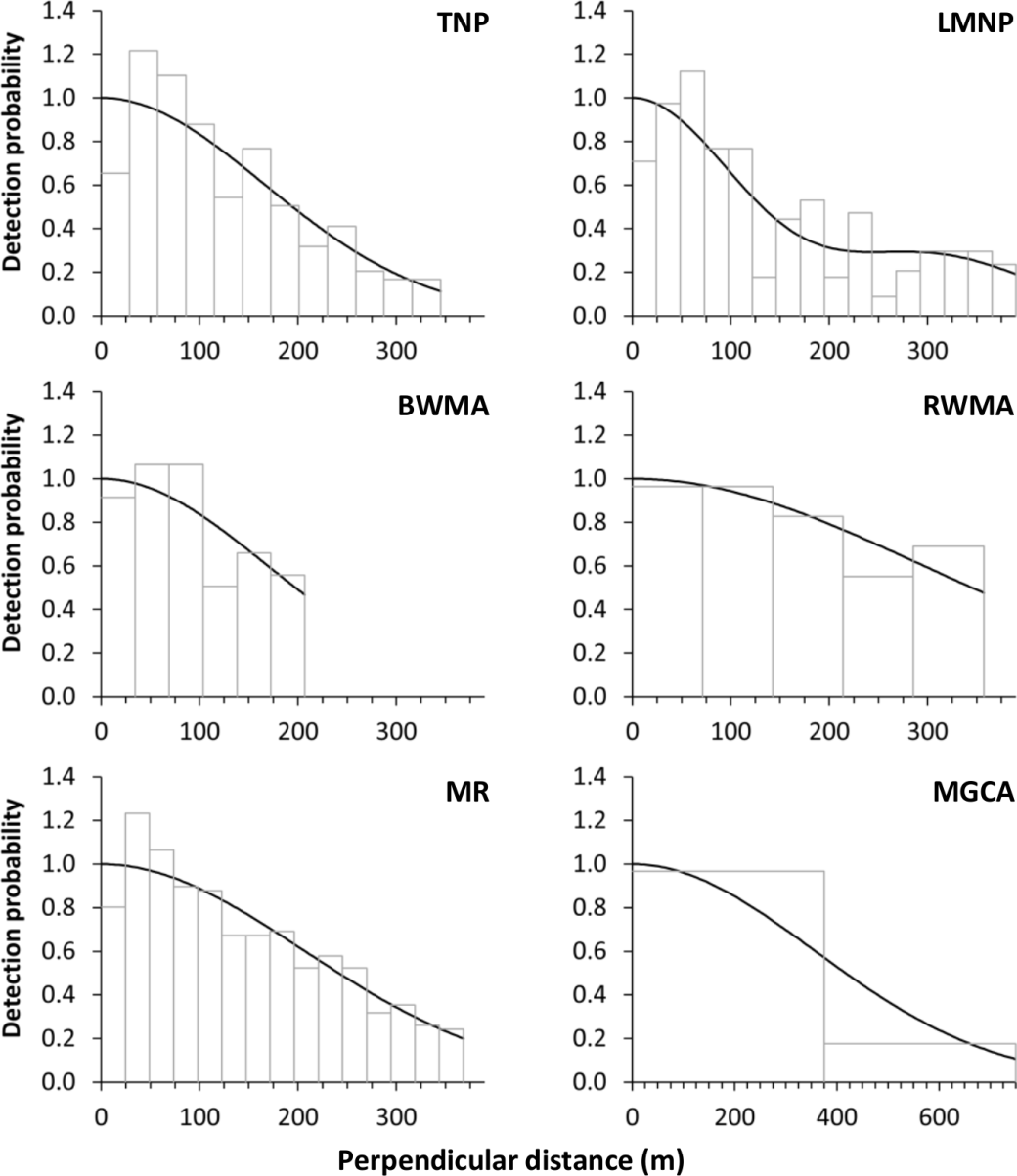


**Fig S3.** Detection functions of zebras in different management units (TNP: Tarangire National Park; LMNP: Lake Manyara National Park; BWMA: Burunge Wildlife Management Area, RWMA: Randilen Wildlife Management Area; MR: Manyara Ranch; MGCA: Mto wa Mbu Game Controlled Area) of the Tarangire Ecosystem, northern Tanzania.


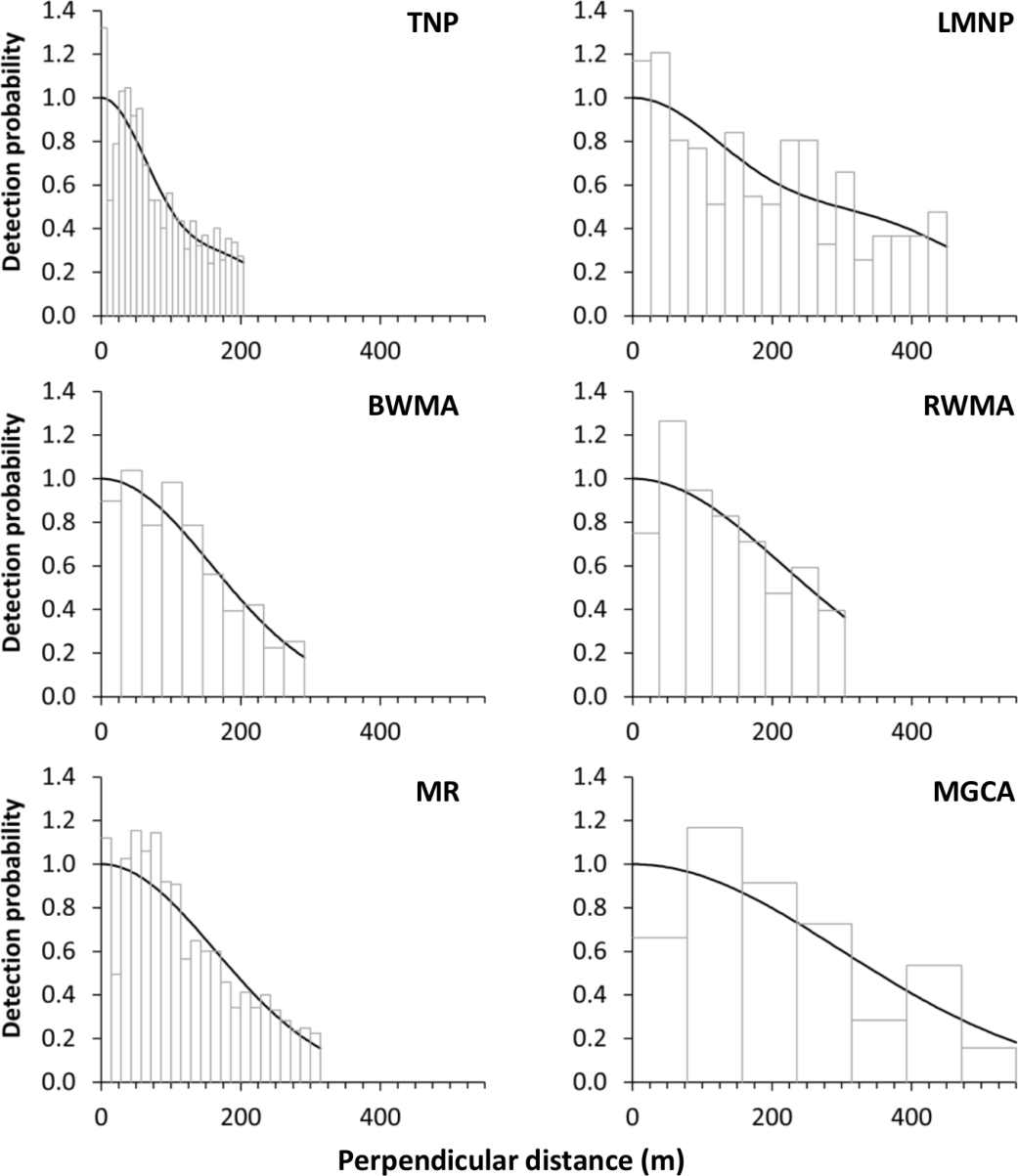


**Fig S4.** Detection functions of wildebeest in different management units (TNP: Tarangire National Park; LMNP: Lake Manyara National Park; BWMA: Burunge Wildlife Management Area, RWMA: Randilen Wildlife Management Area; MR: Manyara Ranch; MGCA: Mto wa Mbu Game Controlled Area) of the Tarangire Ecosystem, northern Tanzania.


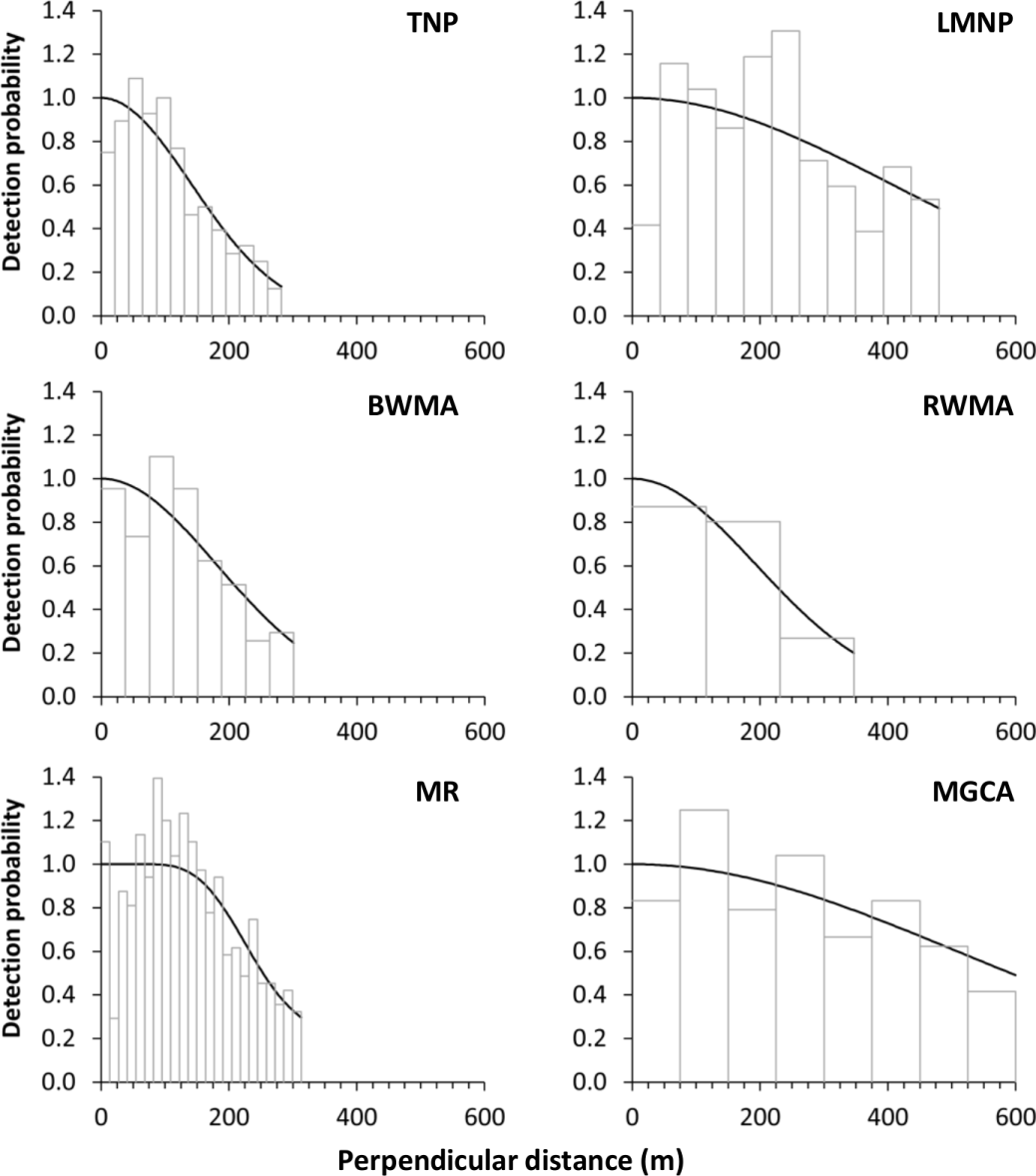

Supplement: Supplementary file 1 — Supplementary Information. [file 41598_2024_66517_MOESM1_ESM.docx]
